# ﻿A new species of *Astronotus* (Teleostei, Cichlidae) from the Orinoco River and Gulf of Paria basins, northern South America

**DOI:** 10.3897/zookeys.1113.81240

**Published:** 2022-07-18

**Authors:** Alfredo Perez Lozano, Oscar M. Lasso-Alcalá, Pedro S. Bittencourt, Donald C. Taphorn, Nayibe Perez, Izeni Pires Farias

**Affiliations:** 1 Instituto de Ciências Biológicas e da Saúde, Universidade Federal de Alagoas (UFAL), Maceió, Brazil Universidade Federal de Alagoas Maceió Brazil; 2 Museo de Historia Natural La Salle, Fundación La Salle de Ciencias Naturales (MHNLS), Caracas, Venezuela Museo de Historia Natural La Salle Caracas Venezuela; 3 Laboratório de Evolução e Genética Animal, Universidade Federal do Amazonas (UFAM), Manaus, Brazil Universidade Federal do Amazonas Manaus Brazil; 4 BioCentro, Universidad Nacional Experimental de los Llanos Occidentales Ezequiel Zamora (UNELLEZ), Guanare, Portuguesa, Venezuela Universidad Nacional Experimental de los Llanos Occidentales Ezequiel Zamora Guanare Venezuela

**Keywords:** DNA, fish, freshwater, morphometrics, osteology, sagitta otoliths, taxonomy

## Abstract

Based on morphological and molecular analysis of *Astronotus* species, a new species is described from the Orinoco River and Gulf of Paria basins in Venezuela and Colombia. Morphologically, it differs from *Astronotuscrassipinnis* and *Astronotusocellatus* in pre-orbital depth, caudal peduncle depth, head width, and caudal peduncle length, with significant differences in average percentage values. Osteologically, it differs from the two described species by lacking a hypurapophysis on the parahypural bone (hypural complex) and having two or three supraneural bones. Another characteristic that helps diagnose the new species is the morphology of the sagitta otolith, which is oval with crenulated dorsal and ventral margins and a rounded posterior edge. Genetically, the new species is distinct from all the other lineages previously proposed for the genus, delimited by five single locus species delimitation methods, and also has unique diagnostic nucleotides. Phylogenetic analyses support the monophyly of the new species as well as all other species/lineages. *Astronotus* species have considerable genetic, anatomical, and sagitta otolith shape differences, but have few significant traditional morphometric and meristic differences, because there is high variability in counts of spines, soft dorsal-fin rays, and lateral-line scales. It is clear that this new species is genetically and anatomically differentiated from all other species within the genus, and deserves recognition as a new valid species.

## ﻿Introduction

The genus *Astronotus* Swainson, 1839 is the only known member of the tribe Astronotini widely distributed in South American river systems (Kullander 1986, 2003). It was originally considered to be a subgenus of *Crenilabrus* Oken, 1817. Kullander (1981, 1983, 1986) reviewed the taxonomy of *Astronotus* and considered only two nominal species as valid: (1) *Astronotusocellatus* (Agassiz, 1831 in Spix and Agassiz 1829). Type locality Atlantic Ocean (error), types in ZSM (lost) distributed in western Amazon and Orinoco basins, with established populations introduced in others states of Brazil as Bahia, Ceará, Espirito Santo, Maranhão, and Piauí states (Burger et al. 2011; Leão et al. 2011; Ramos et al. 2014, 2018; Silva et al. 2020) and outside of South America e.g. Singapore (Ng et al. 1993), Canada (Coker et al. 2001), China (Ma et al. 2003), Hawaii (Mundy 2005), Poland (Solarz 2005), Australia (Hammer et al. 2012; ACTFR 2014), USA (Robins et al. 1991; Fuller et al. 1999; Nico et al. 2019; Rodríguez-Barrera et al. 2020), and Italy (Lorenzoni et al. 2019) and (2) *Astronotuscrassipinnis* (Heckel, 1840). Type locality Rio Paraguay, distributed naturally in southern parts of the Amazon (upper Madeira River) and the upper Paraguay basins (Kullander 1986; Lasso et al. 2001; Kullander 2003; Fricke et al. 2019; Dos Reis et al. 2020) and introduced in Bahia and São Paulo States, and the upper Paraná River in Brazil (Kullander 1981; García et al. 2018; Lopes et al. 2022).

Kullander (1986) reviewed the taxonomy of *Astronotus* species based on morphometric and meristic data from 68 specimens from the Paraguay River basin (Paraná River basin in Paraguay), 18 specimens from several tributaries of the Amazon River basin (Brazil), six specimens from the Orinoco River Basin (Venezuela and Colombia), and 50 from the Peruvian Amazon (Ucayalí and Amazon basins); with this work he redescribed *A.ocellatus* and revalidated *A.crassipinnis*. According to Kullander (2003) both species are medium to large-sized (21–24 cm standard length), and are mainly distinguished by the presence of a group of ocelli (2–12) at the base of the dorsal fin (present in *A.ocellatus* vs. absent in *A.crassipinnis*), the pattern of bars along the sides of the body, and the dorsal-fin spine and ray count (XIII.20 in Peruvian *A.ocellatus* vs. XII.21 in Paraguayan *A.crassipinnis*). However, Colatreli et al. (2012), who molecularly diagnosed the two *Astronotus* species, found that the presence or absence of ocelli is not species-specific and varies between localities and individuals in the same locality. Furthermore, Kullander (1986) found that the meristic characters for the two valid species have significant overlap and are not discriminating.

Kullander (2003) also suggested that there were probably several additional undescribed species in the genus *Astronotus* distributed in the basins of the Amazon, Orinoco, Comté Orapu, Approuague, and Oyapock rivers (French Guiana and State of Amapá, Brazil), and the northern part of the Paraguay River basin. Here we describe a new species of *Astronotus* from the Orinoco River and Gulf of Paria basins by using an integrative taxonomy approach, combining morphometric, meristic, internal anatomy (osteology), otolithometric, and molecular data.

## ﻿Materials and methods

Specimens of *Astronotus* from the Orinoco and Gulf of Pária basins were used for morphological (*n* = 65) and genetic analyses (*n* = 5). To investigate the taxonomic status of *Astronotus* of Venezuela and Colombia, specimens of the two valid species of *Astronotus* were examined: *A.ocellatus* (*n* = 16) and *A.crassipinnis* (*n* = 21). The map was constructed in R 4.1.1 using packages ‘ggspatial’, ‘raster’, ‘rgdal’, ‘rnaturalearth’, and ‘tidyverse’ (R Development Core Team 2011). The final image was edited in Inkscape.

For some specimens, tissue samples were taken from the right side in the posterior region of the flanks and immediately preserved in 98% ethanol. After that, the fishes were fixed in 10% formalin for four weeks and then transferred to 70% ethanol. Specimens were purchased from fishermen or markets, and transported with permits from the Instituto Chico Mendes da Biodiversidade in Brazil (SISBIO N° 54708-1). The fish collection in Venezuela was conducted under a permit to the Universidad Nacional Experimental de los Llanos Occidentales Ezequiel Zamora (UNELLEZ). Voucher specimens are deposited in the collections of The Museo de Ciencias Naturales de Guanare (**MCNG**), Guanare, Venezuela; The Museo de Historia Natural La Salle (**MHNLS**), Caracas, Venezuela; The Museo de Biología de la Universidad Central de Venezuela (**MBUCV**), Caracas, Venezuela; The Estación Biológica Rancho Grande (**EBRG**), Maracay, Venezuela; The Instituto Nacional de Pesquisas da Amazônia (**INPA-ICT**), Manaus, Brazil. Photos of specimens were provided by the Academy of Natural Sciences of Philadelphia (**ANSP**), Pennsylvania, United States of America. Other specimens of *Astronotus* species previously deposited in these collections were also examined. All work was conducted in accordance with the guidelines of the Ethical Committee from the Conselho Nacional de Controle de Experimentação Animal (CONCEA 2016) of Brazil.

### ﻿Morphological methods

**Figure 1.**
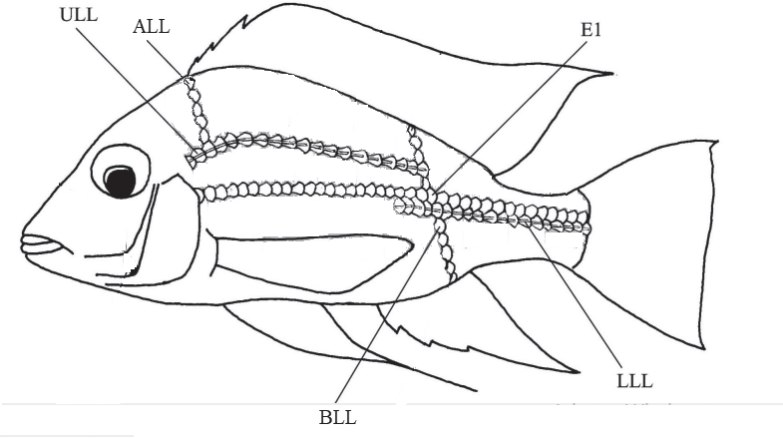
Meristic counts. Abbreviations: ALL - scales above upper lateral line to dorsal fin origin; BLL - scales below lower lateral line to anal-fin; E1 - scales longitudinal above the lower lateral line; LLL - lower lateral-line scales; ULL - upper lateral-line scales. Image modified from López-Fernández and Taphorn (2004).

We followed Kullander (1986, Fig. 1) for morphometric characters and López-Fernández and Taphorn (2004) for meristic characters and scale row nomenclature which consisted of 12 morphometric and ten meristic features, measured using digital calipers (0.10 mm) (Fig. 1). Osteological characteristics of the axial skeleton were analyzed by means of high-definition digital radiographs, in 26 MHNLS specimens of the *Astronotus* of Venezuela and Colombia, using a General Electric X-ray unit, model SenoGrate T 800, processed under the Agfa Viewer NX application. Additionally, and for comparative analysis, ten radiographs of *A.ocellatus* and four of *A.crassipinnis* were analyzed from specimens of the fish collections of INPA, Smithsonian Institution National Museum of Natural History, Washington D.C., U.S.A. (**USNM**) and Zoologische Staatssammlung München, München, Germany (**ZSM**).

The description and denomination of the elements of the axial and caudal skeleton, denominated hypural complex, follow Andreata (1979), Kullander (1983, 1986) and Sibilia and Andreata (1991), with some modifications: preural centrum = **CP**; hemal spine = **HEM**; ural centrum = **CU**; parahypural = **PH**; hypaxial procurrent caudal rays = **HPCR**; hypuraphophysis = **PP**; hypaxial caudal rays = **HCR**; hypurals = **H**; epaxial caudal Rays = **ECR**; diastema = **D**; epaxial procurrent caudal rays = **EPCR**; stegural = **ES**; total caudal rays = **TCR**; epurals = **E**; neural spine = **NEU**. Description of supraneural bones follows Mabee (1988). Description of the jaw teeth and color pattern follow Kullander (1986). All Institutional acronyms follow Sabaj (2016).

**Figure 2.**
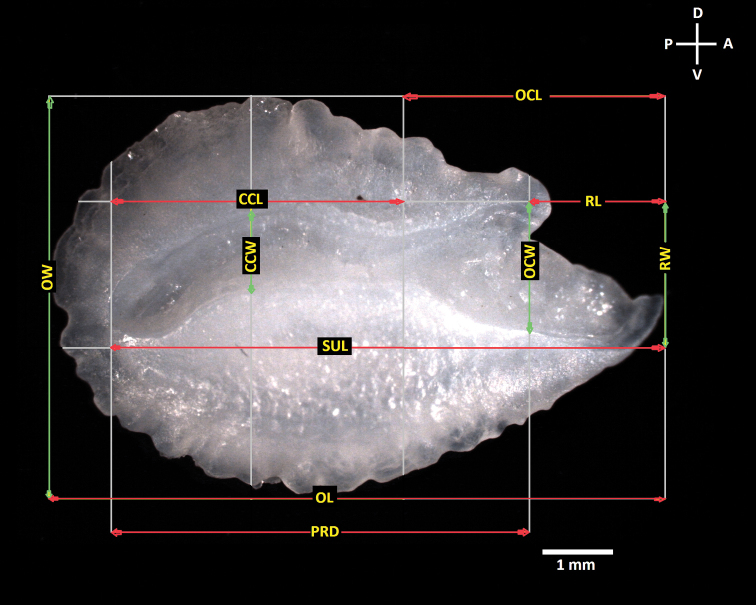
View lateral and internal face of sagitta otolith of *Astronotus*, illustrating measurements. Abbreviations: OL otolith length; SUL - acoustic sulcus length; OCL - ostial colliculum length; CCL - caudal colliculum length; PRD - post rostrum distance; RL - rostrum length; OW - o width; CCW - caudal colliculum width; OCW - ostial colliculum wide; RW - rostrum width.

For the examination of otolith morphology, the sagitta otoliths (*Astronotus* from Venezuela, *n* = 15; *A.ocellatus*, *n* = 10; and *A.crassipinnis n* = 12) were cleaned with a 2% potassium hydroxide solution (10–20 min.) and washed with distilled water. The sagitta otoliths were photographed following the recommendations of AFORO (Lombarte et al. 2006), with a Leica binocular S8-APO stereomicroscope, equipped with a Leica EC3 camera, and processed using Leica Application Suite LAS-EZ digital image analysis software, v. 2.1.0. For the morphological description of the otoliths, the terminology defined by Assis (2000, 2003) and Schwarzhans (1978, 1993) was used (Fig. 2). All measurements of the otoliths were made using the IMAGEJ v. 1.49 software (Rasband 1997–2016; http://imagej.nih.gov/ij/). Otoliths used in this study are deposited in the collection of UFAL and the private collection of AP.

We used the following abbreviations for standard sagitta otolith measurements and ratios: aspect ratio (**Ar**), roundness index (**Rd**), otolith width (**Ow**) morphometric index (**Ow/PRD**). In addition to the standard otolith measurement ratios we also used two new ones: colliculum width-rostrum width ratio (**CCW/RW**), proportion of the CCW of the contained in the RW and rostrum width-post-rostrum distance (**RW/PRD**), proportion of the contained in the PRD as well as standard parameters such as otolith area (**AO**), and otolith perimeter (**PO**), for the calculation of biometric and shape indexes and for the quantitative description of the otoliths (see Suppl. material 1: Table S1 for complete list and descriptions). The differences among the mean values from shape index among species were tested by analysis of variance (ANOVA-one way), using the Paleontological Statistics Software PAST v. 3.0 (Hammer et al. 2001; https://folk.uio.no/ohammer/past). In total, 12 morphometric measurements, nine meristic, and 12 otolithometric were calculated.

To test the discriminant power of biometric and shape indices of the otoliths morphometric data, a Canonical Discriminant Analysis (**CDA**) also was performed using the Paleontological Statistics Software PAST v. 3.0. The CDA orders the groups, maximizing the multivariate variation among the groups in relation to the variance within the group, using Mahalanobis distance as a linear discriminant classifier. The classified data were attributed to the group that resulted with the least distance from Mahalanobis, and the group mean in each group was validated by the jack-knife procedure (Hammer et al. 2001).

### ﻿Molecular methods

Tissue samples collected from 24 individuals (five individuals from Venezuela, seven *A.crassipinnis*, and 12 *A.ocellatus*) were used in molecular analyses. Tissues were preserved in 95% ethanol for DNA extraction and deposited in the Universidade Federal do Amazonas animal tissue collection (CTGA-UFAM). We extracted whole genomic DNA using CTAB (2% CTAB, 1.4 M NaCl, 20 mM EDTA, 100 mM Tris HCl, 1% PVP) extraction protocol plus 15 mg/mL Proteinase K. We amplified 612 bp of the cytochrome C oxidase subunit I (COI) gene via polymerase chain reaction (PCR) using the M13-tailed primer cocktails FishF2/FishR2 and VF2/VR1d (Ivanova et al. 2007) in a total of 15 μL PCR mix, which included 1.5 μL 25 mM MgCl2, 1.5 μL 10 mM dNTPs (2.5 mM each dNTP), 0.5 μL 20 mg/mL Bovine Serum Albumin (BSA), 1.5 μL 10X Taq Buffer with KCl (100 mM Tris-HCl – pH 8.8 at 25 °C – 500 mM KCl, 0.8% (v/v) Nonidet P40), 1.5 μL of primer cocktails (2 pM each), 0.5 μL of Taq DNA polymerase (1 U/μL), 1.0 μL of template DNA (50–100 ng/μL), and 7.0 μL of dd H2O. PCR cycling conditions were as follows: 94 °C (30 s), 35 cycles of 94 °C (30 s), 50 °C (35 s), and 72 °C (90 s), followed by 72 °C (5 min). Exonuclease I – Shrimp Alkaline Phosphatase (ExoSAP) was used to purify PCR products which we then used for fluorescent dye terminator sequencing using primers M13F(-21) and FishR2, following the manufacturer’s recommended protocols for ABI BigDye Terminator (ThermoFisher). We precipitated the sequencing reaction products using 100% Ethanol/125 mM EDTA solution, resuspended it in Hi-Di Formamide, and resolved it on an ABI 3500XL automatic sequencer (ThermoFisher).

We combined all the COI*Astronotus* sequences obtained by Colatreli et al. (2012), with the additional 24 sequences from the present work. We also included 20 sequences obtained in the GenBank database (www.ncbi.nlm.nih.gov/Genbank), with the following accession numbers: *Astronotuscrassipinnis*-JN988692, GU701855, GU701858, GU701859, GU701860, GU701861, GU701862; *Astronotusocellatus*-MG911947, MG911948, MG911949, MH411562, MH411563, MH411564, MH411565; *Cichlaocellaris*-MZ050952, MZ051118, MZ051137, MZ051601, MZ051657, MZ051740. Sequences of *Cichlaocellaris* Bloch & Schneider, 1801 were used as outgroups for subsequent analyses. For the newly collected samples we organized and verified the nucleotide sequences using GENEIOUS 6 (Kearse et al. 2012). The forward and reverse chromatogram reads for each sample sequenced were assembled into Contigs (DNA sequences with overlapping regions, where COI fragments in both ways 5’- 3’ and 3’- 5’ are assembled by similarity) and verified by eye. We then used MAFFT v. 7.07 (Katoh et al. 2002) to perform an automatic alignment using all consensus sequences, followed by a final visual verification. Sequences were also translated into putative amino acids. All new sequences generated in this study are available in GenBank under accession numbers MZ770705–MZ770728. Metadata for all the sequences used in this study are presented in Suppl. material 3: Data S1 (a spreadsheet in xls format) as a flat file following the standard Darwin core format (http://rs.tdwg.org/dwc/terms/index.htm).

### ﻿Molecular and species delimitation analyses

We calculated intra and inter mean genetic distances (uncorrected p-distance, as suggested by Collins et al. (2012)) between the new *Astronotus* species proposed herein and the other two valid species using the packages ape 5.1 (Paradis et al. 2004), and spider 1.4-2 (Brown et al. 2012) of the statistical software R 4.1.1 (R Development Core Team 2011), with the pairwise deletion option set to “TRUE”. Diagnostic nucleotides (Sarkar et al. 2008) for each species/lineage were also delimited using the spider function ‘nuDiag’. A Neighbor-Joining tree containing all sequences is provided as Suppl. material 2: Fig. S1.

For single-locus species delimitation analyses, the total dataset was reduced to a new dataset containing unique haplotypes using the ‘hapCollapse’ function (available at http://github.com/legalLab/protocols-scripts) in the statistical software R. We then generated a Bayesian inference phylogeny using the software BEAST 2.6.2 (Bouckaert et al. 2019) using the following settings: nucleotide substitution model (HKY+I+G) estimated using the BEAST2 package bModelTest 1.2.1 (Bouckaert and Drummond 2017); single site model partition; strict molecular clock; coalescent constant population tree prior. We ran three independent runs with 20 million Markov chain Monte Carlo (MCMC) generations, sampling tree topologies and branch lengths every 2,000 generations, discarding the first 10% generations as burn-in. Convergence between chains was observed by checking the values of effective sample size (ESS > 200) and stationarity of the chain using the software TRACER 1.7.1 (Rambaut et al. 2018). We combined the runs, subsampled at a frequency of 6,000 generations, and burned-in the first 10% generations of each run using LogCombiner (Drummond et al. 2012) to produce a final dataset with 9,000 topologies which were used to produce a maximum credibility tree in TREEANNOTATOR (Bouckaert et al. 2019).

We used the maximum credibility tree as input for five single locus species delimitation analysis: GMYC, the Generalized Mixed Yule Coalescent model (Pons et al. 2006; Monaghan et al. 2009; Fujisawa and Barraclough 2013); bGMYC, a Bayesian implementation of GMYC (Reid and Carstens 2012); ABGD, the Automatic Barcode Gap Discovery (Puillandre et al. 2012); ASAP, Assemble Species by Automatic Partitioning (Puillandre et al. 2021); and LocMin, the local minima method which is a distance threshold optimizing and clustering method implemented in Spider 1.4-2 (Brown et al. 2012); For bGMYC, we used the package splits 1.0–19 (Fujisawa and Barraclough 2013); For ABGD, we used the online web application (https://bioinfo.mnhn.fr/abi/public/abgd/abgdweb.html). For ASAP, we also used the online web application (https://bioinfo.mnhn.fr/abi/public/asap/asapweb.html). All analyses were carried out in the R statistical software and visualized using the package GGTREE (Yu et al. 2017).

## ﻿Results

### ﻿ Astronotus mikoljii sp. nov.

Taxon classificationAnimaliaCichliformesCichlidae

DD831925-2677-5C70-BD45-C7882393CCCE

https://zoobank.org/ECF46E72-25E7-4F1A-A0FC-53581D75241E

Tables 2 , 3 ; Figs 3 , 4 , 5 , 6 , 8a–8 , 9 , 10 , Suppl. materials 1 , 2: Tables S2-S5, Fig. S1

#### Synonymy.

*Astronotusocellatus*. Fowler 1911: 437 (first description with specimens from Venezuela); Luengo 1963: 337 (listed); León 1966: 1127–1134 (brief note); Fernández-Yépez and Antón 1966: 83 (listed); Mago 1967: 259 (listed); Mago 1970a: 76, 78, 80, 87, 88, 92, 96 (biological data), 1970b: 20 (picture, notes), 1978: 14, 15, 17, 22 (picture, brief description); Novoa and Ramos 1978: 134–138 (note, picture); Kullander 1981: 682, 683 (morphological description of Orinoco specimens, distribution); Román 1981: 62, 136 (identification key, picture, note); Cervigón 1982: 354 (picture); Novoa et al. 1982: 312–313, fig. 62 (biological and ecological data, picture); Cervigón 1983: 118–119 (experimental aquaculture); Ginéz and Olivo 1984: 159 (note); Ginéz et al. 1984: 184 (notes); Taphorn and Lilyestrom 1984: 70, 71, 74, 75, 75, 83, 84 (ecological data); Román 1985: 33 (brief morphological description, note, picture); Kullander 1986: 68 (taxonomic status); Novoa 1986: 245 (note); Machado-Allison 1987: 30, 31, 32, 39, 42,43, 47, 48, 60, 61, 62, 94, 114 (biological and ecological data, juvenile picture); Machado-Allison et al. 1987: 136 (listed); Lasso 1988: 371, 372, 381 (list, note); Román 1988: 62 (identification key, picture); Rengifo 1989: 9–16 (note); Winemiller 1989a: 180 (ecological data), 1989b: 241 (ecological data); Novoa 1990: 396 (listed); Rodríguez and Lewis 1990: 322 (listed); Winemiller 1990: 665–672 (ecological data), 1991: 360 (ecological data); Monente 1992: 137 (note); Román 1992: 33 (picture, notes); Machado-Allison 1993: 30, 31, 39, 43, 48, 61, 94, 114 (biological and ecological data, picture); Machado-Allison and Moreno 1993: 83 (note); Royero 1993: 98 (listed); Barbarino-Duque and Taphorn 1994: 100, 101 (note, picture); Winemiller 1996: 111, 112, 129 (ecological data); Winemiller et al. 1996: 26, 38 (biological and ecological data); Rodríguez and Lewis 1997: 114 (ecological data); Fuller et al. 1999: 415 (listed); Lasso et al. 1999: 28 (listed, note); Mojica 1999: 564 (listed, note); Maldonado-Ocampo 2001; 68, 71 (listed, note): Lasso et al. 2003a: 188 (listed, note), 2003b: 244 (listed, note), 2003c: 287 (listed, brief description, note); Machado-Allison 2003: 568 (listed, note); Ajiaco-Martínez et al. 2012: 144 (listed); Herrera et al. 2012: 65 (listed); Machado-Allison et al. 2013: 305, 307, 332 (listed, note); Ortega-Lara 2016: 81 (picture, listed); Ramírez-Gil and Ajiaco-Martínez 2016: 131 (table 3: listed); DoNascimiento et al. 2017: 99 (listed, note); Winemiller et al. 2018: 5, 6 (listed, ecological data).

Astronotuscf.ocellatus. Lasso et al. 1999: 28 (listed); Lasso and Machado-Allison 2000: 56, 57, 154 (diagnostic features, type locality, note, picture); Lasso et al. 2003b: 244 (listed, note), 2003c: 287 (listed, taxonomic note); Lasso 2004: 377,378 (diagnostic, features, biological and ecological data); Taphorn et al. 2005: 24, 30, 32, 34 (listed, notes); Medina and Bonilla 2006: 3, 4, 6, 9 (picture, genetic data); Marcano et al. 2007: 46 (listed); Brito et al. 2011: 310 (diagnostic features, note, picture); Echeverría and Machado-Allison 2015: 81, 83 (listed), Machado-Allison et al. 2018: 442, 443, 454, 455, 483 (picture, painting, morphologic description, biological and ecological data).

*Astronotus* sp. Lasso et al. 2003a: 188 (ecological data); Rodríguez-Olarte et al. 2003: 205, 211 (listed ecological data); Antonio and Lasso 2004: 92, 93, 107 (diagnostic features, comparative material, note); Campo 2004: 59 (listed); Hoeinghaus et al. 2004: 88, 92 (listed, notes); Lasso et al. 2004: 148 (listed, note); López-Fernández et al. 2005: 646, 649–651 (morphological characters); Galvis et al. 2007: 272, 394 (diagnostic features, note, picture); Lasso et al. 2009a: 119 (listed, note), 2009b: 143 (listed, note), 2010: 57, 71 (listed, brief description, note); Machado-Allison et al. 2010: 223 (listed); Lasso et al. 2011a: 64 (listed, note), 2011b: 102–108 (listed, identification key); Villa-Navarro et al. 2011: 277 (listed); Machado-Allison et al. (2013): 305, 307, 332 (listed, notes); Lasso et al. 2014: 103 (note, picture); Usma et al. 2016: 117 (listed).

#### Type material.

**Figure 3.**
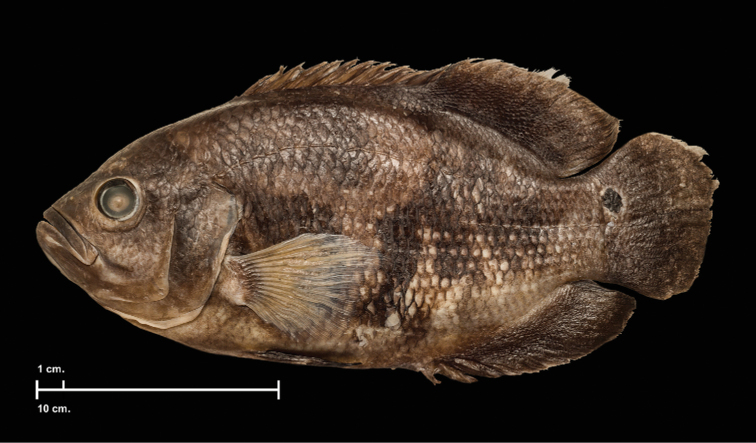
*Astronotusmikoljii* sp. nov., preserved holotype MCNG 56677 (240.12 mm SL), Venezuela., Estado Apure, Municipio Pedro Camejo in a small stream tributary of Arauca River. Photograph: Ivan Mikolji.

***Holotype*.**MCNG 56677 (225.1 mm SL), Venezuela. Estado Apure, Pedro Camejo Municipio in a small stream (tributary of the Arauca River), 07°33'14.08"N, 67°38'44.06"W, 13 Jun 2015, Pérez A. and Alfonso R. leg. (Fig. 3; Tables 1, 2).

***Paratypes*.**MCNG 56678 (5, 175.3–200.4 mm SL); MBUCV 35750 (1,144.4 mm SL); MHNLS 26123 (1, 140.4 mm SL); INPA-ICT 057800 (2, 112.3–143.4 mm SL); EBRG 11061 (1, 152.2 mm SL), Venezuela, Estado Apure, Municipio Pedro Camejo, small tributary stream of the Arauca River, same date and collectors as holotype; Venezuela, ANSP 37896 (1, 124.5 mm SL). Estado Monagas, Las Piedritas Caño Uracoa, 24.9 km SW of Uracoa, 8°48'9.00"N, 62°28'25.00"W, 12 Feb 1911, Bond F. and Brown S. leg.; MHNLS 198 (5, 97.5–116.7 mm SL) Estado Apure, Achaguas, Río Apure, 7°55'35"N, 68°28'47"W, 10, Jan 1951, Fernández-Yépez A. leg.; MHNLS 3551 (2, 109.1–121.9 mm SL). Estado Anzoátegui Laguna de Mamo, 1 km south of Juasiullal, right bank of the Orinoco River, 7°25'57"N, 63°7'8"W, 22 Jan 1981, Feo G., Pérez L., Ovidio H. leg.; MHNLS 3776 (3, 98.7–115.9 mm SL) Estado Bolivar, Orinoco River, Fajardo Island Main channel, May 05, 1975, Köpke H.; MHNLS 3777 (1, 119.1 mm SL) Estado Bolivar, Orinoco River, Fajardo Island Main channel, 19 Apr 1974, Köpke H. leg.; MHNLS 4850 (1, 79.1 mm SL) Estado Bolivar, Rio Claro Lagoon, approximately 15 Km East of San Félix, 02 Apr 1986, Pérez L. leg.; MHNLS 4914 (1, 121.7 mm SL) Estado Bolivar, Residual Lagoon, Hato Puga; approximately 25 km East of San Felix, 11 Apr 1986, Pérez L. leg.; MHNLS 4915 (1, 121.6 mm SL) Estado Bolivar, Laguna Chirere approximately 30 km west of Puerto Ordáz, 04 Apr 1986, Pérez L. leg.; MHNLS 4916 (1, 117.3 mm SL) Estado Bolivar, Laguna Río Claro approximately 15 km East of San Felix, 07 Apr 1987, Lasso C. Pérez L. leg.; MHNLS 7860 (2, 94.0–107.5 mm SL) Estado Cojedes, El Baul, 8°54'48.60"N, 68°17'17.52"W, 15 Apr 1984, B. Román leg.; MHNLS 9022 (1, 85.3 mm SL) Estado Bolivar, Caicara del Orinoco, Rio Aripao to Chaviripa River, 26 Mar 1986, B. Román leg.; MHNLS 9050 (1, 125.6 mm SL) Estado Guárico, Esteros de Camaguán, 6 May 1984, B Román leg.; MHNLS 11719 (1, 118 mm SL) Estado Apure, Caño Guaritico Hato El Frío, 7°52'35.00"N, 66°55'57.00"W, 18 Jan 1991, Lasso-Alcalá O. Lasso C. leg.; MHNLS 13094 (1, 201.5 mm SL) Estado Bolivar, Laguna Patiquín floodplain Caño Mato, tributary of Caura River, 7°9'16.00"N, 65°11'57.00"W, 23 Mar 1998, Vispo, C. leg.; MHNLS 13812 (2, 108.7–119.3 mm SL) Estado Delta Amacuro, Orinoco River Delta, Caño Ibaruma, Serranía de Imataca, 8°1'0.00"N, 60°47'0.00"W, 24 Jan 2003, Ponte V. leg.; MCNG 3608 (1, 148.9 mm SL) Estado Apure, Modules of UNELLEZ area adjacent to south of dike Caño Caicara, 7°25'30.00"N, 69°32'20.00"W, 2 Jun 1981, Donald Taphorn leg.; MCNG 2522 (1, 106.6 mm SL) Estado Apure, 3.4 km south of Bruzual bridge west side of road, 8°01'20.00"N, 69°20'50.00"W, 17 Nov 1980, Donald Taphorn leg.; MCNG 1832 (1, 118.5 mm SL) Estado Apure, 3.4 km south of Bruzual bridge 8°01'20.00"N, 69°20'50.00"W, 15 Nov 1980, Donald Taphorn leg.; MCNG 5993 (1, 101.3 mm SL) Estado Apure, Hato el Frio, 30 Sep 1979, Craig Lilyestrom leg.; MCNG 4994 (3, 193.1–210.1 mm SL) Estado Apure, Modules Fernando Corrales (UNELLEZ) dike east, 7°29'30.00"N, 69°31'W, 27 Nov 1981, Donald Taphorn leg.; MCNG 32543 (1, 101.6 mm SL) Estado Barinas, culvert 10 km NW of Libertad on road to Barinas 8°20'3.00"N, 69°43'41.00"W, 25 Jan 1995, John Armbruster leg.; MCNG 1675 (4, 197.4–240.7 mm SL) Estado Barinas, borrow pit at end of runway at Arismendi, 8°29'50.00"N, 68°21'20.00"W, 14 Sep 1980, Donald Taphorn leg.; MCNG 32596 (1, 161.9 mm SL) Estado Barinas, Río Caipe, to the east of town La Luz, 8°24'31.00"N, 69°48'19.00"W, 26 Jan 1995, John Armbruster leg.; MCNG 26930 (2, 112.50–104.90 mm SL) Estado Cojedes, San Geronimo in Hato Santa Clara, 02 Nov 1991, Manuel Gonzalez Fernandez leg.; MCNG 789 (2, 185.6–193.4 mm SL) Estado Delta Amacuro, Caño Paloma Orinoco River Delta, 21 Feb 1978, John Lundberg leg.; MCNG 32033 (1, 195 mm SL) Estado Guárico, P.N. Aguaro-Guariquito, Río Aguaro at El Paso to Médano Gómez, 7°50'27.00"N, 66°30'23.00"W, 01 Nov 1995, Donald Taphorn leg.; MCNG 32006 (2, 152.2 mm SL) Estado Guárico, P.N. Aguaro-Guariquito, Río Aguaro in Laguna Begonia, 7°52'6.00"N, 66°30'36.00"W, 01 Nov 1995, Donald Taphorn leg.; MCNG 25187 (1, 183.3 mm SL) Estado Guárico, Calabozo highway, Camaguán, 14 Jan 1982, Otto Castillo leg.; MCNG 32739 (1, 180.1 mm SL) Estado Guárico, P.N. Aguaro-Guariquito, Laguna Médano Gómez, 06 Aug 1995, Aniello Barbarino-Duque leg.; MCNG 11580 (2, 101.8–122.3 mm SL) Estado Guárico, borrow pit in savannah 2.3 km from San Fernando de Apure between km 305 and 306, 7°55'20.00"N, 67°28'20.00"W, 22 Mar 1981, Donald Taphorn leg.; MCNG 26815 (3, 90.5–109.0 mm SL) Estado Portuguesa, Caño Maraca, 8°53'11.00"N, 69°29'18.00"W, 13 Jan 1992, Larry Page, Pat Ceas, Brooks Burr, Steve Walsh, Chris Taylor, Leo Nico, Kirk Winemiller leg.; MCNG 15461 (1, 127.4 mm SL) Estado Portuguesa, Brazo del Caño Maraca at ranch of Darío Urriola, 26 Oct 1984, Kirk Winemiller leg.; MCNG 9097 (3, 112.3–138.1 mm SL) Estado Portuguesa, borrow pit N of Moritas east of Guanare-Las Moritas road, 8°45'30.00"N, 69°34'30.00"W, 03 Jan 1979, Donald Taphorn leg.; MCNG 5740 (1, 110.6 mm SL) Estado Portuguesa, Caño Maraca at bridge via Guanarito, Km 60, 8°49'50.00"N, 69°20'20.00"W, 28 Aug 1980, Donald Taphorn leg.; MCNG 29650 (1, 112.3 mm SL) Estado Portuguesa, Caño San José between Guanarito and La Capilla, 8°41'9.00"N, 68°56'49.00"W, 01 Mar 1994, John Armbruster leg.; Colombia • MHNLS 23575 (1, 114.7 mm SL), Departamento de Vichada, Caño drainage of Laguna Cajaro right bank of the Guaviare River, 3°58'14.00"N, 67°59'8.00"W, 16 Feb 2008; C. Lasso M. Sierra. M. Patiño F. Villa. A. Ortega. leg.; MHNLS 24053 (1, 218 mm SL), Departamento de Vichada, Caño Vitina right bank affluent, Rio Inírida upstream from Caranacoa beach, 3°44'30.00"N, 67°56'10.00"W, 21 Feb 2008, C. Lasso. M. Sierra M. Patiño F. Villa A. Ortega. leg.; MHNLS 24054 (1, 227.5 mm SL), Departamento de Vichada, Caño Vitina tributary right bank Río Inírida, upstream from Playa de Caranacoa, 3°44'30.00"N, 67°56'10.00"W, 21 Feb 2008, C. Lasso M. Sierra M. Patiño F. Villa; A. Ortega leg.; MHNLS 24059 (1, 205.5 mm SL), Departamento de Vichada, Peluame Lagoon, left Bank Guaviare River, near Guaviare-Inírida Confluence, 3°57'51.00"N, 67°55'W, 17 Feb 2008, C. Lasso M. Sierra M. Patiño F. Villa A. Ortega; S. Usma leg.; MHNLS 24061 (1, 211.5 mm SL), Departamento de Vichada, Laguna Bolívar, left bank, flooded area, Orinoco River, between Guanayana and Amanaven farms, 4°7'N, 67°45'W, 26 Feb 2008, C. Lasso M. Sierra M. Patiño F. Villa leg.; MHNLS 24064 (1, 226 mm SL), Departamento de Vichada, Caño Vitina, tributary right bank Río Inírida, upstream of Playa de Caranacoa, 3°44'30.00"N, 67°56'10.00"W, 21 Feb 2008, C. Lasso M. Sierra M. Patiño F. Villa; A. Ortega leg.

**Table 1.** Comparison of morphometric data from *Astronotusmikoljii* sp. nov., *A.ocellatus* and *A.crassipinnis*. The measurements are expressed in mm; all other measurements are expressed as percentage of SL as mean (*X*); standard deviation (± SD), and range (min – max).

Morphometric variable	*A.mikoljii* sp. nov.		*A.crassipinnis*		*A.ocellatus*	
	*X* (± SD)	(min–max)	*X* (± SD)	(min–max)	*X* (± SD)	(min–max)
Head length (H)	36.72 ±1.85	(31.78–42.76)	35.01 ±1.25	(32.44–36.75)	33.26 ±1.65	(30.50–36.50)
Snout length (snout)	11.53 (±1.23)	(9.09–14.86)	5.36 (±0.85)	(4.12–6.97)	10.67 (±0.67)	(9.18–1.73)
Body depth (body)	46.5 (±3.43)	(39.18–53.57)	51.26 (±1.86)	(49.03–55.50)	46.19 (±3.36)	(40.38–52.29)
Orbital diameter (O)	9.06 (±1.09)	(7.25–12.35)	7.36 (±0.64)	(6.14–8.36)	7.73 (±1.18)	(6.02–11.32)
Head width (HW)	21.83 (±1.11)	(19.49–25.41)	21.53 (±1.32)	(19.65–23.78)	19.64 (±3.31)	(8.13–23.25)
Inter-orbital width (Int-Orb)	13.8 (±1.11)	(12.2–17.89)	14.19 (±1.39)	(11.49–16.78)	14.79 (±2.00)	(13.32–21.89)
Pre-orbital depth (Pre-Orb)	14.22 (±1.88)	(11.09–18.06)	10.14 (±1.13)	(8.62–13.02)	15.91 (±1.47)	(13.98–18.72)
Caudal peduncle depth	17.29 (±1.05)	(15.01–20.14)	17.1 (±0.62)	(15.65–18.22)	16.45 (±1.13)	(14.43–18.41)
Caudal peduncle length	10.32 (±2.17)	(7.41–19.79)	11.09 (±0.57)	(10.02–12.17)	12.81 (±0.74)	(11.63–14.44)
Pectoral-fin length (P1)	29.29 (±3.03)	(23.02–36.15)	30.03 (±2.33)	(24.51–34.47)	29.66 (±1.78)	(27.38–33.22)
Pelvic-fin length (P2)	23.34 (±4.45)	(16.63–34.73)	23.49 (±2.51)	(19.38–27.85)	24.22 (±4.71)	(17.21–33.97)
Last dorsal spine length	9.92 (±2.40)	(6.46–15.46)	9.72 (±1.78)	(7.24–12.59)	7.59 (±1.65)	(5.07–12.18)

**Table 2.** Comparison of meristic data from *Astronotusmikoljii* sp. nov., *A.ocellatus*, and *A.crassipinnis*.

Meristic variable	*A.mikoljii* sp. nov.	*A.crassipinnis*	*A.ocellatus*
	mode (min–max)	mode (min–max)	mode (min–max)
Dorsal-fin rays (D)	20 (17–21)	18 (16–24)	18 (17–21)
Anal-fin rays (A)	18 (16–20)	18 (15–21)	17 (16–20)
Longitudinal scales (E1)	38 (35–41)	35 (33–41)	33 (31–35)
Upper lateral line scales (ULL)	20 (18–21)	21 (19–22)	19 (18–22)
Lower lateral line scales (LLL)	18 (15–21)	16 (12–20)	13 (11–16)
Scales above lateral line (ALL)	7 (7–8)	7 (7–8)	6 (6–7)
Scales below lateral line (BLL)	10 (6–12)	12 (11–14)	12 (12–13)
Circumpeduncular scales (SPC)	28 (26–32)	30 (26–31)	29 (27–30)
Ceratobranchial gill rakers	10 (9–11)	9 (9–12)	11 (10–11)
Opercular scales	3 (3–5)	4 (3–5)	4 (3–5)
Cheek scales	8 (7–11)	10 (7–11)	11 (7–11)

#### Comparative material.

*Astronotusocellatus*. NPA-ICT 026472 (1) Brazil, Amazonas, Catalão, rio Solimões Bacia do Solimões, 3°9'34.00"S, 59°54'44.00"W, 20 Dec 2002; INPA-ICT 050911 (1) Brazil, Amazonas, rio Solimões, Ilha da Paciência, 3°20'5.60"S, 60°12'11.30"W, Manaquiri, 18 Dec 2011; J. Santos, R. Orta, F. Pena leg.; INPA-ICT 050912 (1) Brazil, Amazonas, rio Solimões, Ilha da Paciência, 3°20'5.60"S, 60°12'11.30"W, Manaquiri, 18 Dec 2011; J. Santos, R. Ota, F. Pena leg.; INPA-ICT 033076 (1) Brazil, Tabatinga, rio Solimões, 3°57'32.00"S, 69°20'19.00"W, town of Palmares, 02 Sept 2003; Jansen Zuanon leg.; INPA-ICT 033913 (1) Brazil, Amazonas, São Sebastião de Uatumã, rio Uatumã, 30 Oct 2009; R. Leitão, R. Lazzarotto leg.; INPA-ICT 033889 (1) Brazil, Amazonas, rio Nhamunda, 2°13'51.00"S, 56°46'23.00"W, município Nhamunda, 21 Sept 2009; R. Leitão, R. Lazzarotto leg.; INPA 050452 (2) Brazil, Amazonas, rio Preto da Eva, 2°44'38.10"S, 59°28'38.60"W, highway AM-010, km 110, 20 Aug 2014; INPA 22331 (1) Brazil, Amazonas, Lago do Boto, RDS do lago Piranha, Manacapuru, 30 Jan 2003; Ivanildo; INPA-ICT 033437 (2) Brazil, Amazonas, Lago Ressaca Grande, rio Solimões, 2°28'26.00"S, 66°9'17.00"W, Fonte Boa, 08 Sept 2003; Jansen Zuanon leg.; INPA 17486 (1) Brazil, Amazonas, pool in Lago Secado, rio Purus, Santa Lucia, 03 Jun 2001; Lucia Rapp-Daniel leg.; INPA 17364 (3) Brazil, Amazonas, Lago Campinas, rio Purus, 05 Jun 2001; Lucia Rapp Py-Daniel leg; INPA-ICT 029312 (49) Brazil, Amazonas, RDS Uacari, stream near community of Pupunha; 5°35'47.00"S, 67°47'13.00"W; 26 Nov 2007; Martins, A.R. leg.; INPA-ICT 007143 (1) Brazil, Para, Rio Cupari, near mouth of Tapajon River, 3°44'31.00"S, 55°23'25.00"W, 27 Oct 1991; Zuanon, J.A. leg.; INPA-ICT 007170 (1) Brazil, Para, Rio Cupari, near mouth of Tapajos River; 27 Oct 1991; Zuanon, J.A. leg; INPA-ICT 007333 (1) Brazil, Tocantins, Rio Tocantins, Icangui; Brazil, Pará, Tucuruí, 3°49'49.80"S, 49°38'21.84"W, 28 Jun 1980; Equipe de Ictiologia do INPA leg.; INPA-ICT 020454 (1) Brazil, Tocantins, Lago das Ariranhas, rio Araguaia; Brazil, Tocantins, Caseara, 9°14'5.28"S, 49°57'59.76"W, 11 Nov 2000; Equipe de Ictiologia do INPA leg.; INPA-ICT 020663, (2) Brazil, Tocantins, Rio Tocantins, Jabutizão, 7°43'55.56"S, 49°28'31.80"W, 10 May 2000; Santos, G.M. leg; INPA-ICT 040585 (1) Brazil, Pará, Mercado do Porto, collected from stream tributary to Xingu River, near Vitória do Xingu, 2°52'51.00"S, 52°0'45.00"W, 23 Sept 2013; Sabaj, M. H. leg; INPA-ICT 043339 (6) Brazil, Pará, Xingu River, specimens bought in market, near Tucuri stream around Vitória do Xingu; 07 Mar 2014; Martins, A.R. leg.

*Astronotuscrassipinnis*. INPA-ICT 021697 (1) Brazil, Rondônia, rio Novo, Guaporé, 11°29'29.00"S, 64°34'34.00"W, 27 Jul 2003; Torrente Vilara; INPA-ICT 038549 (2) Lago do Bodo, Bom Jardim, Porto Velho (Brazil, Rondônia), 8°32'31.00"S, 63°37'26.00"W, 12 Ago 2011, L. Costas, F. Viera, leg.; INPA-ICT 049921 (3) Brazil, Rondônia, Rio Guaporé, Surpresa, 10°06'11"S, 65°38'44"W, 21 Set 1985; G.M. dos Santos leg.; INPA-ICT 049922 (2) Brazil, Rondônia, mouth of Guaporé River, near Surpresa, 11°19'44"S, 64°60'11"W, 16 Jun 1984; Costa Marques, G.M. dos Santos leg.; INPA-ICT 049923 (1) Brazil, Rondônia, Rio Pacaás-Novos, blackwater flooded forest ca. 15 km upstream from mouth of Pacaás Novos River, Guajará-Mirim, 02 Apr1987, G.M. dos Santos leg.

#### Diagnosis.

**Figure 4.**
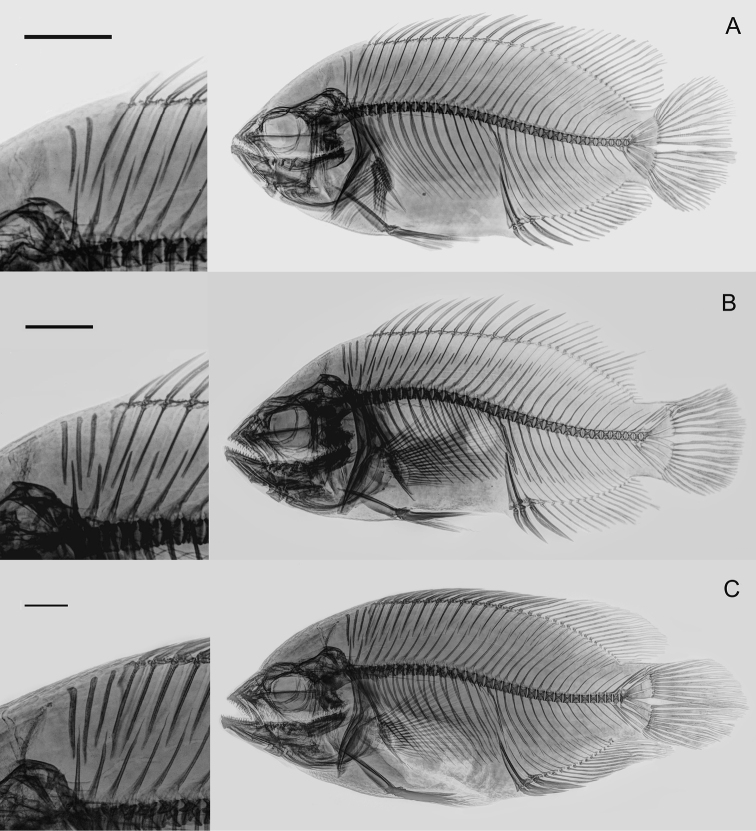
Radiographs of paratypes of *Astronotusmikoljii* sp. nov. and details of the supraneural bones **A**MHNLS 198 (116.7 mm SL) **B**MHNLS 26123 (140.4 mm SL) **C**MHNLS 24054 (227.5 mm SL). Scale bars: 10 mm.

**Figure 5.**
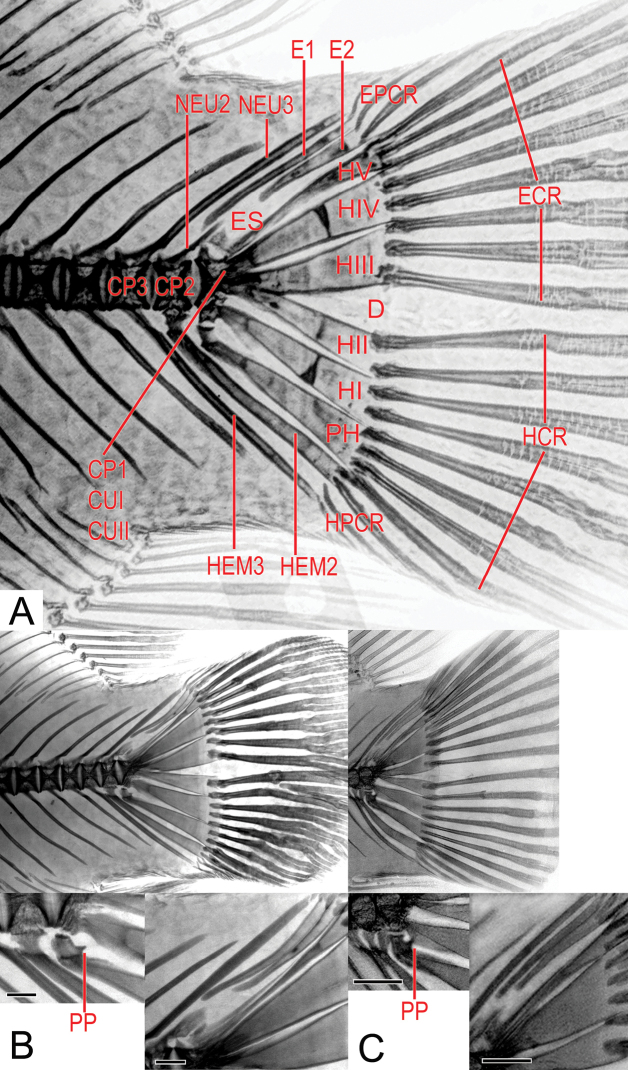
Radiographs of the caudal skeleton in *Astronotus* species showing magnified details **A***A.mikoljii* sp. nov. (MHNLS 24059, 205.5 mm SL) **B***A.crassipinnis*INPA-ICT 33889 (204.3 mm SL) **C***A.ocellatus*USNM 284442 (79.6 mm SL). Abbreviations: hypurapophysis (PP) of parahypural bone (PH), epurals (E1 – E2) and neural spine (NEU2, diastema (D), preural centrum (CP), epaxial caudal rays (ECR), hypaxial caudal rays (HCR), epaxial procurrent caudal rays (EPCR) hypaxial procurrent caudal rays (HPCR). Scale bars: 2 mm.

**Figure 6.**
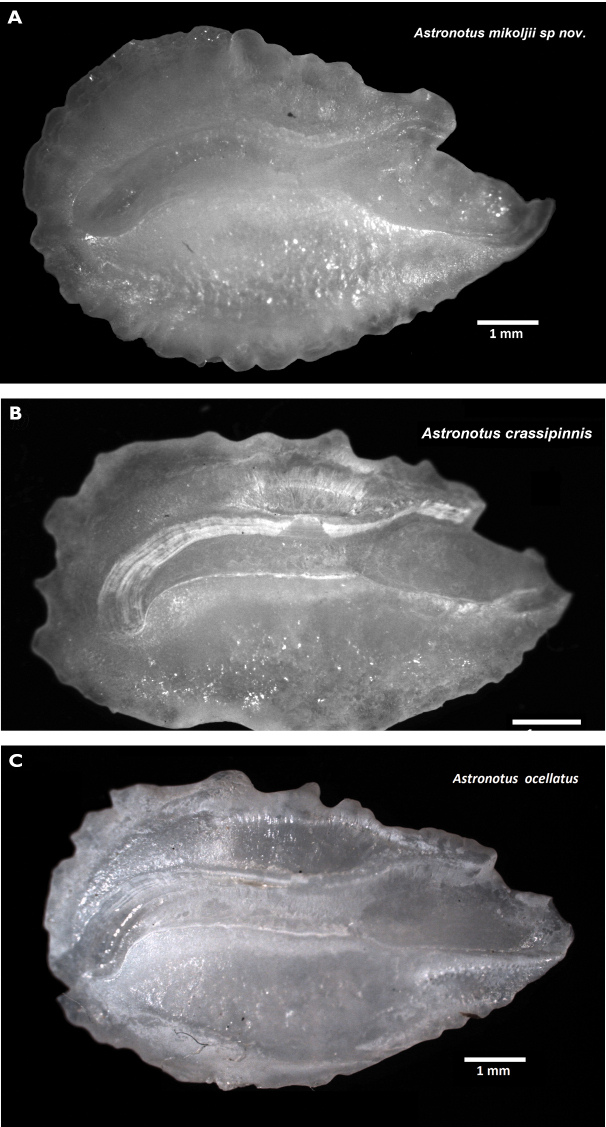
Left sagitta otoliths (medial view) of **A***Astronotusmikoljii* sp. nov. **B***A.crassipinnis***C***A.ocellatus.* Scale bars: 1 mm.

The new species is distinguished from congeners by the following combination of characters: two or three supraneural bones (Fig. 4) (vs. two); absence of the spinous process (hypurapophysis) on the anterosuperior border of the parahypural bone (hypural complex) in *Astronotusmikoljii* sp. nov. (vs. present in *A.ocellatus* and *A.crassipinnis*) (Fig. 5). The sagitta otolith in *A.mikoljii* sp. nov. is oval, with strongly crenulated ventral and dorsal margins (vs. elliptical and smooth-lobed margins in *A.crassipinnis*, and elliptical and smooth-dentate margins *A.ocellatus*); the rostrum is projected with an elongated process, in *A.mikoljii* sp. nov. (vs. rostrum process short in *A.crassipinnis* and *A.ocellatus*); the posterior region of the sagitta otolith is rounded in *A.mikoljii* sp. nov. (vs. straight or flat in *A.crassipinnis* and *A.ocellatus*) (Fig. 6). The aspect ratio of sagitta otoliths in *A.mikoljii* sp. nov. (AR = 0.665) is higher than that of *A.ocellatus* (AR = 0.606), and *A.crassipinnis* (AR = 0.585), and the differences are statistically significant at *P* < 0.05. The roundness index was highest in *A.mikoljii* sp. nov. (Rd = 0.597) vs. *A.ocellatus* (Rd = 0.545) and *A.crassipinnis* (Rd = 0.543) (*P* < 0.05). Also the morphometric index showed higher values in *A.mikoljii* sp. nov. compared to *A.ocellatus* (0.837 vs. 0.767) and *A.crassipinnis* (0.735) (Suppl. material 1: Table S2). The new species also is distinguished from congeners by the following combination of morphometric characters: the mean head length of *A.mikoljii* sp. nov. (36.72% SL) is longer than that of *A.crassipinnis* (35.01% SL), and also *A.ocellatus* (33.26% SL); the mean diameter of the orbit of *A.mikoljii* sp. nov. (9.06% SL) is greater than that of *A.ocellatus* (7.36%SL) and that of *A.crassipinnis* (7.73% SL); the mean pre-orbital depth of *A.mikoljii* sp. nov. (14.22% SL) is greater than that of *A.crassipinnis* (10.14% SL) but less than that of *A.ocellatus* (15.91% SL); the mean snout length of *A.mikoljii* sp. nov. (11.53% SL) is longer than that of *A.crassipinnis* (5.36% SL), and *A.ocellatus* (10.67% SL) (Tables 1, 2).

#### Description.

***Morphology*.** Morphometric and meristic data are presented in Table 3. Body moderately oval; laterally compressed, widest at region of anterior flank and posterior part of head; Dorsal-fin base contour sloping from about middle of spinous portion. Caudal peduncle edges horizontal; ventral sometimes longer than dorsal. Head and snout short; orbit slightly below forehead contour, entirely in upper and anterior halves of head. Interorbital wide, slightly convex. Tip of exposed maxilla extending to anterior edge of orbit; lower jaw articulation below middle of orbit. Both lip folds interrupted, junction of upper and lower lips African type. Opercula and pectoral girdle bones smooth. Interorbital convex; pre-pelvic contour straight; greatest body depth at pelvic-fin bases.

**Table 3.** Morphometric and meristic data (mm) of holotype and paratypes of *Astronotusmikoljii* sp. nov., with specimen number (n); mean (*X*); standard deviation (SD); variation coefficient (CV); minimum value (Min); maximum value (Max).

Morphometric variable (mm)	Holotype	Paratypes (*n* = 65)							
		*X*	SD		CV		Min		Max
Standard length	240.12	134.61	42.42		31.52		79.11		240.12
Head length	76.50	48.91	13.75		28.12		31.07		81.70
Snout length	21.90	15.47	4.67		30.20		9.72		29.10
Body depth	110.40	64.50	19.86		30.79		35.58		110.40
Orbital diameter	17.80	11.90	2.56		21.56		9.40		18.43
Head width	47.80	29.76	9.18		30.88		17.07		49.54
Inter-orbital width	30.90	18.94	6.63		35.04		10.98		37.47
Pre-orbital depth	26.70	18.85	6.33		33.62		12.44		39.22
Caudal peduncle depth	36.30	23.35	7.15		30.62		13.27		41.54
Caudal peduncle length	26.40	13.95	5.57		39.94		6.32		26.40
Pectoral-fin length	57.20	39.74	11.36		28.59		25.22		69.51
Pelvic-fin length	46.90	31.82	8.63		27.13		18.90		51.29
Length of last dorsal spine	15.90	13.35	4.520		33.84		8.30		28.40
**Meristic variable**	**Holotype**	**Paratypes (*n* = 65)**							
				**Min**		**Max**		**mode**	
Dorsal-fin rays (D)	XIII,18			XII,18		XIV, 20		XIII, 19	
Anal-fin rays (A)	III,18			III,14		III, 20		III, 16	
Pectoral-fin rays (P1)	15			14		17		15	
Pelvic-fin rays (P2)	I,5			I,5		I,5		I,5	
Caudal-fin rays (C)	20			19		24		22	
Longitudinal scales (E1)	37			35		42		38	
Upper lateral line scales (ULL)	20			18		21		20	
Lower lateral line scales (LLL)	17			16		21		18	
Scales above lateral line (ALL)	7			7		8		7	
Scales below lateral line (BLL)	9			6		12		10	
Circumpendicular scales (SPC)	28			26		32		28	
Opercular scales	4			3		5		3	
Cheek scales cheek	9			7		9		8	
Ceratobranchial gill rakers	10			8		11		10	
Pre-dorsal midline scales	16			14		18		16	
Tubed scales in lower lobe caudal	5			1		8		6	

***Scales*.** Pre-dorsal midline scales irregularly arranged, ca. 14–18 along midline; posterior pre-pelvic scales about half size of flank scales, slightly smaller anteriorly, in ca. seven horizontal series. Scales around caudal peduncle 26–32; lower lobe of caudal fin with 1–8 tubed lateral-line scales, from base to middle usually with gaps between them, and from half to edge of fin continued by pored scales). Anterior 1/3 to 1/2 of the cheek naked, remainder with cycloid scales; cheek scale rows 3 (*n* = 65; range 7–9). Operculum covered with eight cycloid scales (*n* = 65; range 3–5); opercula scales in ca. four vertical series, sub-opercular scales in two or three series: inter-operculum with one or two scales close to pre-opercular corner and six or seven scales in principal series. Pre-operculum naked. Soft unpaired fins covered by dense scale layer. Spinous dorsal fin bordered by posteriorly progressively wider scale layer with straight margin. This basal scale layer continued onto basal 1/3 of soft dorsal fin but inter-radial scales distal to it widen scaly layer to basal 1/2 of fin medially. Pectoral and pelvic fins naked. Inter-pelvic squamation extended laterally to cover bases. Caudal fin completely scaled save for narrow zone along hind margin; basal scales ctenoid; inter-radial scales cycloid in three or four series between rays.

***Fins*.** One continuous dorsal fin, with anterior portion of hard rays (spines) and posterior portion with soft rays. First dorsal-fin spine inserted slightly in advance of vertical from hind margin of operculum; relative length of spines increasing to 4^th^ then subequal to last few which are longer, twice length of first or slightly longer. Soft part of dorsal fin with rounded tip, reaching to not quite middle of caudal fin or to 3/4 of caudal fin. D. XII.18 (3), XII. 19 (4), XII. 20 (5), XIII. 17 (5), XIII. 18 (8), XIII. 19 (14), XIII. 20 (12), XIII. 21 (5), XIV. 18 (4), XIV. 19 (5), XIV. 20 (3); Anal-fin origin opposite soft dorsal-fin origin; soft portion similar to soft dorsal fin, but not reaching beyond middle of caudal fin. A. III. 14 (5), III. 15(10), III. 16 (15), III. 17 (10), III. 18 (14), III. 19 (3), III. 20 (1). Pectoral-fin with blunt dorsal tip, 4^th^ ray longest, hind margin truncate or slightly curved; sometimes reaching to first anal-fin spine P1. 15 (*n* = 65; range 14–17). Pelvic-fin spine inserted below pectoral axilla; fin pointed, with outer branch of first ray longest, reaching to first anal-fin spine to 1/3 of soft-anal fin base, inner rays gradually shorter P2, 1.5 (1.5). Caudal fin with hind edge rounded, with 22 (*n* = 65; range 19–24), total rays (Table 3).

***Gills*.** First gill arch with rudimentary denticles exposed laterally, two or three on epibranchial, one in angle, and 8–11 on ceratobranchial. Tiny gill-rakers present externally on medial side short, compressed and heavily denticulate (Table 3).

***Teeth*.** Lower jaw with two teeth rows on each side (external and internal). External tooth row in both jaws extends from tip to end of each bone (dentary and maxilla). Teeth in outer series stout, conical, pointed, little recurved; anterior three or four in each jaw half as strong as rest; outer series to near end of upper jaw (20) and of corresponding length in lower jaw; inner band of very small weak teeth, less than 0.4 mm long, only anteriorly in jaws.

***Otoliths*.** Sagitta otoliths oval with crenulate posterior, dorsal, ventral margins; Ar was greater than 0.66, otolith Rd 0.59 (Suppl. material 1: Table S2). Anterior region (rostrum) projected with elongated process and rounded posterior region. Anti-rostrum short and rounded, moderately broad ostium incisure with notch. Acoustic canal (sulcus acoustics) heterosulcoid, ostial, medial; ostium rectangular and shorter than caudal colliculum, which is tubular, closed, and strongly curved along its posterior margin.

***Dorsal and vertebral skeleton*.** Pre-caudal vertebrae 15, caudal vertebrae 17, and total vertebrae 32. Range in vertebral counts (pre-caudal, caudal, and total) is wide (14–16, 15–18, 30–33). Two or three supraneural bones present, first anterior to neural spine of first pre-caudal vertebrae, second and third, between that spine and second neural spine of second pre-caudal vertebrae (Fig. 4, Suppl. material 1: Table S3).

***Caudal skeleton*.** Includes hypural complex and 20–24 caudal rays. This complex has five vertebral elements, CP1, CUI, and CUII or urostyle (all fused), CP2 and CP3. This last element has HEM3 that can support one or two HPCR and a NEU3 that can be free or support up to two EPCR. The CP2 is articulated with HEM2, which can be free or articulated with up to two HPCR or one or two HC = R. Likewise, CP2 on its upper side almost converges with bone E1 that is free or articulated with EPCR or ECR. The complex CP1 + CUI + CUII, is articulated on its lower side with four elements, PH and HI, which are articulated with two to four HCR each, the HII, which is articulated with one or two HCR and the HIII, which can support two or three ECR. Complex CP1 + CUI + CUII articulated on its anterior side with bone HIV, which in turn can support two to five ECR. On its upper side this complex is articulated with the ES bone that is fused with HV and can support between one to three ECR. Above HV, and always separated from Complex CP1 + CUI + CUII, E2 is positioned, which can be found without rays or an EPCR or ECR. Next and always separated from the E2, E1 is found which along its upper side only supports an EPCR and on the lower edge may be articulated and even fused with NEU2. Finally, NEU3 is observed, originating along the upper edge of CP3, which can be free or articulated with up to two EPCR (Fig. 5, Suppl. material 1: Table S4).

***Color in alcohol*.** The background color varies from dark yellow to dark brown; chest color varies from pale to dark brown; abdomen whitish. Operculum and cheek pale brown. Snout and forehead chestnut. Sides of the body with irregular vertical bars (chestnut or pale brown) sometimes difficult to see, of different widths, individually variable. Sometimes with pattern of 1–3 pale and dark vertical bars, normally with pale, lambda-shaped bars; central part of these bars is usually divided at level of abdomen, forming lambda (λ) figure with bases extending to pelvic fins. Dorsal and anal fins pale or dark brown with paler edges on both. Caudal fin dark brown, darker on base, always with black ocellus surrounded by narrow white or grey ring, placed in superior part of caudal-fin base, and marginally extending onto caudal peduncle. Pectoral and pelvic fins hyaline. Dorsal fin without rings or ocelli (Fig. 3).

***Color in life*.** Sexual dimorphism not observed. Ventrum pale grey, chest dark grey, abdomen whitish, operculum and cheek grey to brown, snout and forehead chestnut, underside of head dark grey with greyish or greenish tinge over chestnut. Sides of body with barely visible irregular vertical bars (chestnut or dark grey) of different widths and patterns, which may vary from one individual to another. Wide vertical bar of dark brown color crosses central part of body and reaches spinous portion of anal fin. Central part of said bar usually divided at level of abdomen, forming lambda (λ) shape with bases extending to pelvic fins. Posterior side of the body with abundant iridescent orange spots that can appear longitudinally. Dorsal and anal fins dark brown with paler edges. Caudal fin dark grey, darker on base. Always with black ocellus surrounded by orange or yellow ring that reaches center of lateral line of caudal fin and extends onto caudal peduncle. Pectoral fins hyaline, dorsal and pelvic fins without spots or ocelli (Fig. 8a).

#### Molecular analysis.

We amplified 612 bp of the COI gene for the 24 *Astronotus* specimens used for genetic analyses. The addition of sequence data (58 sequences) of Colatreli et al. (2012) and the sequences obtained in GenBank (20 sequences) increased this dataset to 102 specimens. This alignment was then reduced to a total of 22 unique haplotypes of *Astronotus* plus two haplotypes of *Cichlaocellaris* as outgroups. Sequence length varied from 468 to 664 bp, with a mean sequence length of 626 bp; 23 sites were parsimony-informative. No indels were observed. No internal stop codons were found. All *Astronotus* species (*A.ocellatus*, *A.crassipinnis*, and *A.mikoljii* sp. nov.) and additional suggested distinct lineages (*Astronotus* sp. “Jurua”, *Astronotus* sp. “East”, and *Astronotus* sp. “Negro”) shows reciprocal monophyly in the maximum credibility tree, with high posterior probability support (≥ 0.95) (Fig. 9).

All five single-locus species delimitation methods delimited *Astronotusmikoljii* sp. nov. as a distinct lineage and, overall, the only discrepancy between the methods occurred in the method bGMYC, which identified *A.crassipinnis* and *Astronotus* sp. “East” as a single lineage. The maximum intraspecific distance within *A.mikoljii* sp. nov. was 0.163%, while minimum inter-specific distance was 0.98% (Table 4). The lineage *Astronotus* sp. “Negro” is the closest lineage to *A.mikoljii* sp. nov. The mean genetic distance between *A.mikoljii* sp. nov. and the currently valid species (*A.crassipinnis* and *A.ocellatus*) had values of 1.75% and 2.15%, respectively (Table 5). A total of three diagnostic sites segregates *A.mikoljii* sp. nov. from *A.ocellatus* and *A.crassipinnis* (Table 4).

**Table 4.** Max intra/inter-specific distances, Nearest Neighbor, and diagnostic nucleotides between *Astronotus* delimited lineages and species.

Species/Lineage	max_intra (%)	min_inter (%)	Nearest Neighbor	Diagnostic nucleotides									
				89	272	395	447	512	539	578	596	662	total
***A.mikoljii* sp. nov.**	0.163	0.98	***Astronotus* sp. “Negro**”	g	**T**	**A**	c	**G**	a	g	c	t	3
***A.crassipinnis***	0.388	0.904	***Astronotus* sp. “East**”	g	c	g	c	a	**G**	g	**A**	**C**	3
***A.ocellatus***	0.546	0.753	***Astronotus* sp. “Jurua**”	g	c	g	**T**	a	a	g	c	t	1
***Astronotus* sp. “East**”	0.301	0.753	***A.crassipinnis*, *Astronotus* sp. “Negro**”	g	c	g	c	a	a	**A**	c	t	1
***Astronotus* sp. “Jurua**”	0.151	0.753	***A.ocellatus***	g	c	g	c	a	a	g	c	t	0
***Astronotus* sp. “Negro**”	0.301	0.753	***Astronotus* sp. “East**”	**A**	c	g	c	a	a	g	c	t	1

**Table 5.** Mean inter-specific distances between *Astronotus* delimited lineages and species.

Mean_inter (%)	*A.mikoljii* sp. nov.	*A.crassipinnis*	*A.ocellatus*	*Astronotus* sp. “East”	*Astronotus* sp. “Jurua”	*Astronotus* sp. “Negro”
***A.mikoljii* sp. nov.**	-	2.15	1.75	1.36	2.09	1.04
***A.crassipinnis***	2.15	-	2.2	2.15	0.92	2.08
***A.ocellatus***	1.75	2.2	-	1.03	2.51	1.32
***Astronotus* sp. “East**”	1.36	2.15	1.03	-	2.07	0.97
***Astronotus* sp. “Jurua**”	2.09	0.92	2.51	2.07	-	1.78
***Astronotus* sp. “Negro**”	1.04	2.08	1.32	0.97	1.78	-

#### Multivariate analysis.

The Canonical Discriminant Analysis (CDA) using morphometric data of the sagitta otoliths clearly identified three groups corresponding to each of the described species of the genus *Astronotus* (Fig. 10). A high level of successful classification among the species was obtained using the jack-knife procedure, reaching a value ± 90% in both cases (Suppl. material 1: Table S5). In this statistical analysis, including the measurements on the geometric shape of the sagitta otoliths separated *A.mikoljii* sp. nov. from *A.ocellatus* and *A.crassipinnis*.

#### Etymology.

The specific name is given to honor Mr. Ivan Mikolji, Venezuelan explorer, artist, author, underwater photographer, and audiovisual producer, in recognition for being a tireless and enthusiastic diffuser of the biodiversity and natural history of freshwater fishes, conservation of aquatic ecosystems of Venezuela and Colombia, and for logistic support for this work. Since 2020, Ivan Mikolji has been recognized as Associate Researcher of the Museo de Historia Natural La Salle, from the Fundación La Salle de Ciencias Naturales, in Caracas, Venezuela.

#### Distribution.

**Figure 7.**
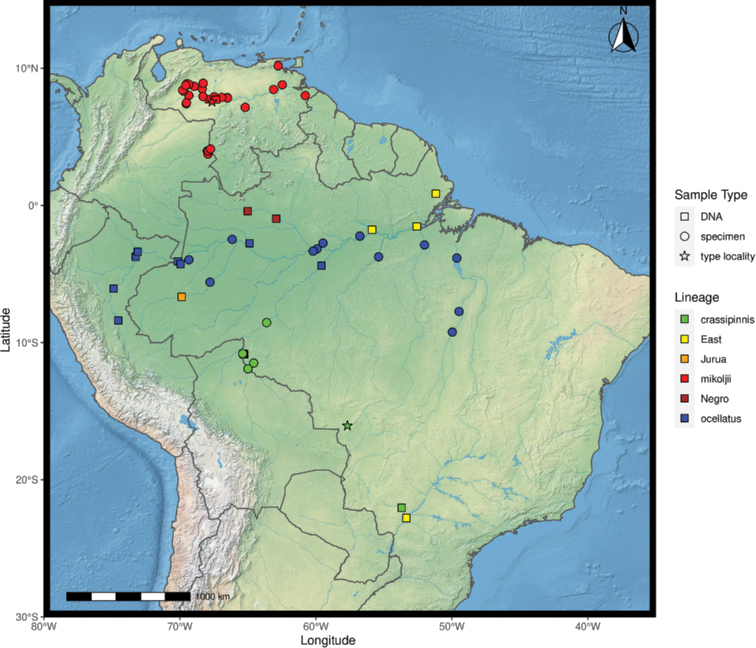
Map showing all the sites sampled in this study. The circles represent sampling localities based on specimen records used for morphological analyses, the squares represent sampling localities of the genetic material analyses, and the stars represent the type locality of each species. The colors represent the consensus of the species delimitation methods. *Astronotusmikoljii* sp. nov. (red), *A.crassipinnis* (green), *A.ocellatus* (blue), *Astronotus* sp. “East” (yellow), *Astronotus* sp. “Jurua” (orange), and *Astronotus* sp. “Negro” (brown).

*Astronotusmikoljii* sp. nov. is distributed in all parts of the lower Orinoco River basin (Fig. 7), along the floodplain of its main channel and in the drainages of the following rivers (or sub-basins): Atabapo, Inírida, Guaviare, Vichada, Bita, Meta, Tomo, Arauca, Apure, Caura, Morichal Largo and Delta, in Venezuela and Colombia (Fowler 1911; Novoa and Ramos 1978; Kullander 1981; Novoa et al. 1982; Román 1985; Novoa 1986; Román 1988; Winemiller 1989a, b, 1990; Lasso and Castroviejo 1992; Monente 1992; Machado-Allison 1993; Lasso et al. 1999; Mojica 1999; Ponte et al. 1999; Lasso and Machado-Allison 2000; Lasso et al. 2003a, b, c, 2004; Campo 2004, Lasso 2004; Machado-Allison 2003; Antonio and Lasso 2004; Taphorn et al. 2005; Galvis et al. 2007; Marcano et al. 2007; Lasso et al. 2009a, b; Brito et al. 2011; Lasso et al. 2011a, b; 2014; Echeverría and Machado-Allison 2015; Ortega-Lara 2016; DoNascimiento et al. 2017; Machado-Allison et al. 2018; Winemiller et al. 2018). It also occurs in the Gulf of Paria basin (Caño La Brea, Forest Reserve of Guarapiche, sub-basin San Juan River, EBRG 5055) in Venezuela (Lasso et al. 2010). It has been introduced in other watersheds of Venezuela such as Lago de Valencia and in reservoirs of the Mar Caribe basin (drainages of the Unare, Tuy, Coro and San Juan rivers (Isla de Margarita)) (Luengo 1963; León 1966; Cervigón 1983; Ginéz and Olivo 1984; Ginéz et al. 1984).

#### Ecology.

**Figure 8.**
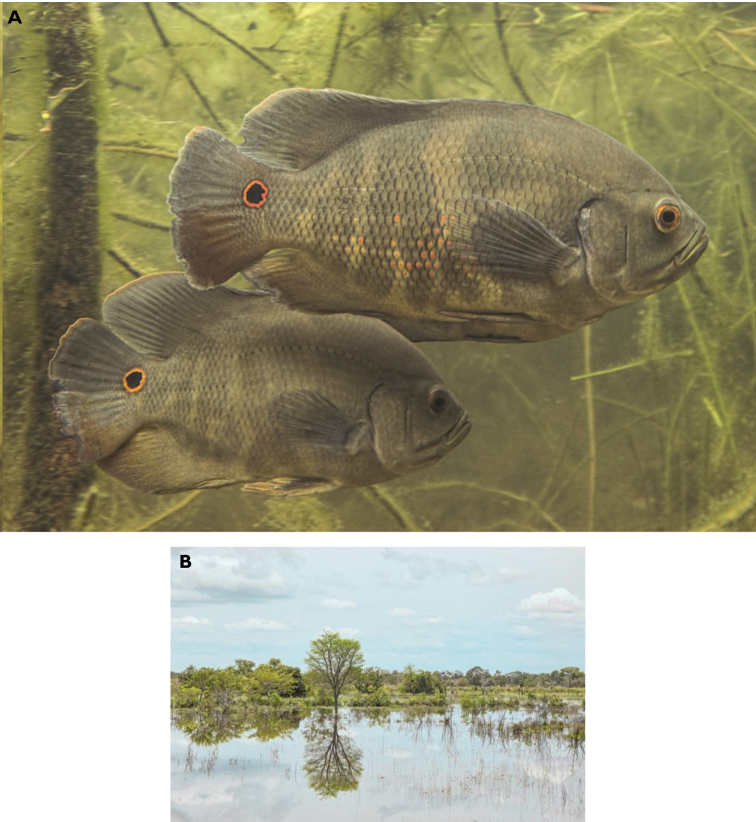
*Astronotusmikoljii* sp. nov. **A** live coloration of specimens collected with holotype **B** Natural shallow pond and type locality in floodplain of Arauca River Venezuela. Photographs: Ivan Mikolji.

*Astronotusmikoljii* sp. nov. usually inhabits the middle and lower reaches of the Orinoco River and the Gulf of Paria basin, at altitudes not exceeding 250 m a.s.l. (Fig. 8b). It can be found in either lotic or lentic water bodies, large or small rivers, culverts, lagoons, and floodplains, with white, clear, and black waters (sensu Sioli 1965). In the Orinoco River Delta, it lives in slow-flowing channels and flooded forests, while in the middle Orinoco region; it has only been captured in flood and floodplain lagoons, on both banks of the river (Novoa et al. 1982; Novoa 1986). In its first stages of development, it is associated with floating vegetation and semi-rooted plants formed mainly in grasses and water hyacinth (*Paspalumrepens*, *Eichhorniacrassipes*). In the adult phase they are located in riparian zones, generally among grasses and sedges (Machado-Allison 1993; Lasso 2004; Lasso et al. 2011b; Echeverría and Machado-Allison 2015). Part of the type material was captured in the flooded savannah of a minor tributary of the Arauca River, in the floodplains of Apure State, Venezuela (Fig. 8b). During sampling, the body of water was almost stagnant and the water temperature was 27 °C with abundant aquatic vegetation and muddy bottom.

This species was probably negatively impacted by the invasion of the Orinoco River Basin by transferred invasive cichlid *Caquetaiakraussii* (Steindachner, 1878) (Royero and Lasso 1992; Señaris and Lasso 1993; Lasso and Machado-Allison 2000). For example, in one lagoon in the Portuguesa River drainage between Guanare and Guanarito, where one of the authors (DCT) commonly collected *A.mikoljii* sp. nov. (on many occasions over many years during student field trips from 1978 to 1988) it is now absent, having been completely replaced by *C.kraussii*.

#### Common names.

In Spanish and indigenous local languages, names which are known for *Astronotusmikoljii* sp. nov. in Venezuela are pavona, vieja, cupaneca, Oscar, mijsho (Kariña), boisikuajaba (Warao), hácho (Pumé = Yaruro), phadeewa, jadaewa (Ye’Kuana = Makiritare), perewa, parawa (Eñepá = Panare), yawirra (Kúrrim = Kurripako), kohukohurimï, kohokohorimï, owënawë kohoromï” (Yanomami = Yanomamï) (Barandiarán 1962; Mago 1967, 1970c; Novoa et al. 1982; Obregón et al. 1984; Román 1985; Novoa 1986; Román 1988; Bedoya 1992; Mattei-Müller et al. 1994; Lasso and Machado-Allison 2000; Mosonyi 2002; Machado-Allison 2003; Vispo and Knab-Vispo 2003; Mattei-Müller and Serowe 2007; Brito et al. 2011) and pavo real, carabazú, Oscar, mojarra, mojarra negra, eba (Puinave), Itapukunda (Kurripako), uan (Tucano) in Colombia (Sánchez 2008). The suggested common name for this species in the aquarium hobby is “Mikolji’s Oscar” in English, “Oscar de Mikolji ‘’ in Spanish.

## ﻿Discussion

Through an integrative taxonomic approach, our results demonstrate the distinctiveness of *Astronotusmikoljii* sp. nov. from *A.ocellatus* and *A.crassipinnis*, using genetic characters (DNAm), skeletal characters (presence of two or three supraneural bones and absence of spinous process in upper border of parahypural bone of caudal fin), and anatomical characters (sagitta otolith shape). Although sagitta otolith morphology has not been previously used as a diagnostic character in *Astronotus*, as shown in the CDA, each species of *Astronotus* has a differently shaped, distinct sagitta otolith that can be used to distinguish not only the new species, but also between *A.ocellatus* and *A.crassipinnis*.

**Figure 9.**
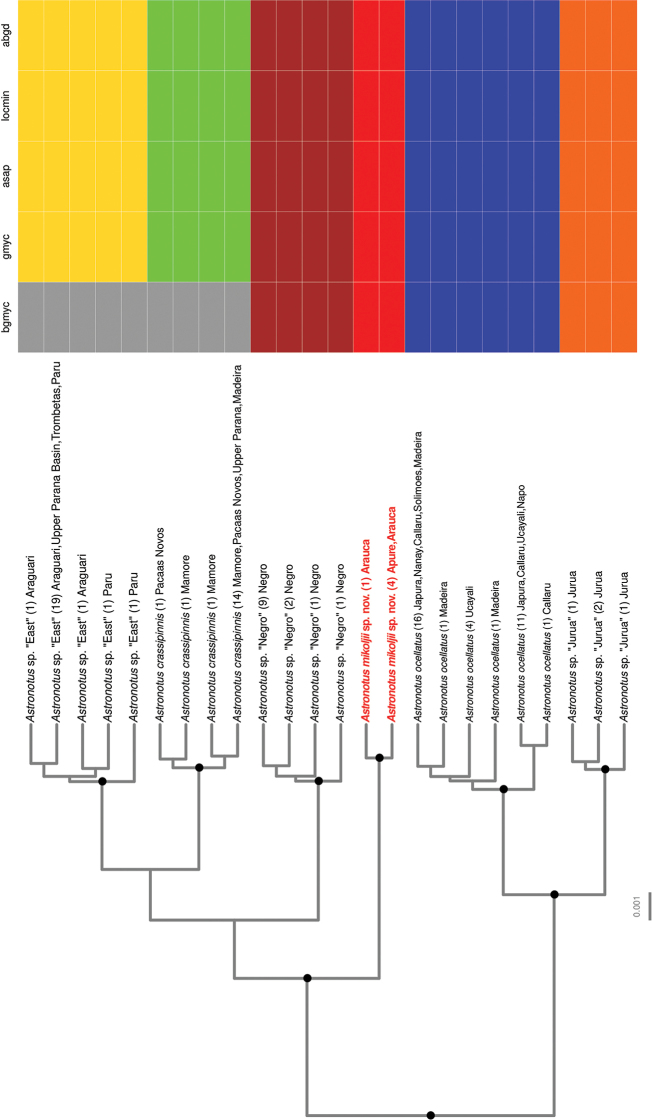
Maximum clade credibility tree from 9,000 posterior trees generated using BEAST 2.6. Dataset comprised 22 unique haplotypes (from a total of 102) of *Astronotus*COI sequences. Bayesian posterior probabilities above 0.95 are shown as dark nodes. Species delimitations are shown by method as colored boxes. The number of collapsed individuals is indicated in parentheses and outside of it the locations where they were sampled. The scientific name of the new species is red. The figure was created in R 4.1.1 using the package ‘ggtree’ and the final graphic in Inkscape.

**Figure 10.**
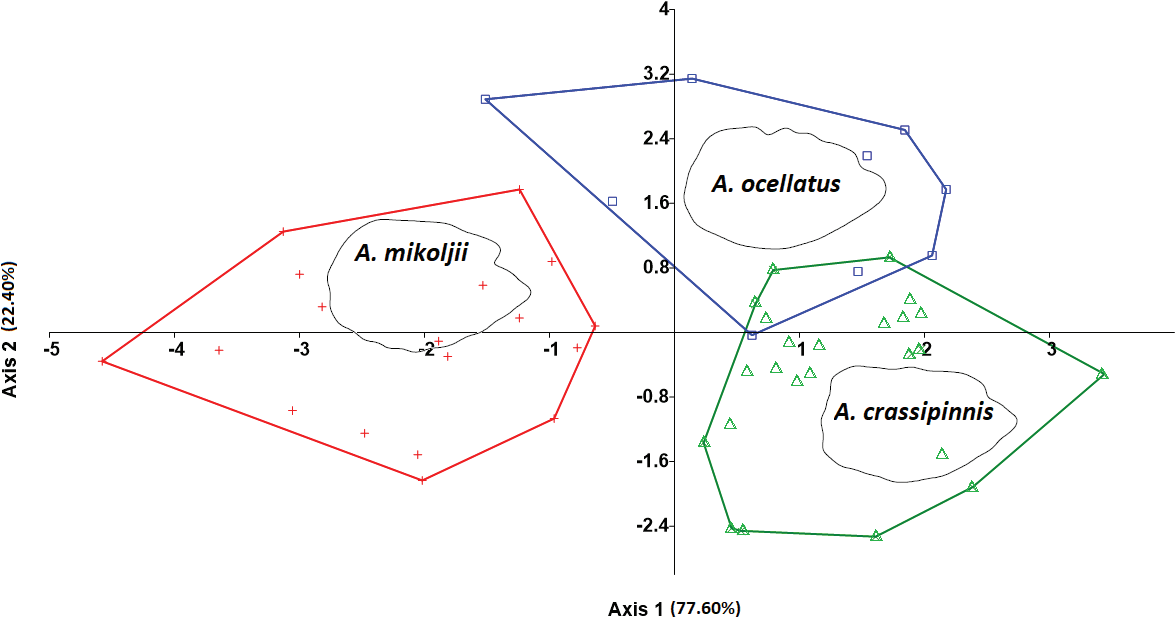
Plot of scores of Canonical Variants Analysis (CVA), from comparative sagitta otoliths morphometric data of *Astronotusmikoljii* sp. nov. (red crosses), *A.ocellatus* (blue squares), and *A.crassipinnis* (green triangles).

The molecular analyses also show significant differences among *Astronotusmikoljii* sp. nov. *A.crassipinnis*, and *A.ocellatus*, in addition to identifying that all lineages/species are reciprocally monophyletic in mitochondrial DNA (Fig. 9), with mean genetic p-distance of 2.15 and 1.75% respectively (Table 4). Although *A.mikoljii* sp. nov. can be diagnosed by otolithometrics and molecular data (Figs 9, 10), its differentiation by morphometry and body meristics shows little promise. Comparison of *A.ocellatus* specimens from the western and Central Amazon, as well as specimens of *A.crassipinnis* from Madeira rivers basins with *A.mikoljii* sp. nov., revealed the difficulty in distinguishing these three species using external morphometric characters. Only four morphometric characters showed significant differences (head length, snout, orbital diameter and pre-orbital distance) between the three species.

Other characteristics of the axial skeleton anatomy also support the validity of the new species. The presence of up to three supraneural elements in at least 30% of the examined x-ray specimens (*n* = 26) of *Astronotusmikoljii* sp. nov. (Fig. 4b, c, Suppl. material 1: Table S3), is highly unusual and distinctive. The supraneural bones, formerly known as predorsal spines, are a character of great importance in the phylogenetic classification of Teleost fishes (Fink and Fink 1981; Mabee 1988). In some groups of the family Cichlidae, the number of supraneural bones has been used to separate genera and species (Gosse 1975; López-Fernández and Taphorn 2004; López-Fernández et al. 2006; Sparks and Stiassny 2010; Arbour and López-Fernández 2011; Martinez et al. 2015). According to our review, it is not common to find members of the Cichlidae family in America that have three supraneural bones, since most of the Neotropical species have only two or sometimes just one element (e.g., Kullander 1986, 1998; López-Fernández et al. 2005; Kullander and Ferreira 2006; Chakrabarty and Sparks 2007). However, Kullander (1986), when describing the genus *Bujurquina*, indicates that it presents exceptional cases with one or three elements that represent abnormal conditions, in which the third supraneural seems to be a pterygiophore without spines. In our case, without doubt, the third element in *A.mikoljii* sp. nov. is clearly a supraneural bone, clearly separated from the first pterygiophore (Fig. 4b, c). Wijkmark et al. (2012) apparently found a specimen (28 specimens had only two elements) of *Andinoacarablombergi* with three supraneural bones, but they do not present any supporting illustration. The presence of such a high number of supraneural elements is best known in Ostariophysan fishes (Fink and Fink 1981; Chuctaya et al. 2020; Stiassny et al. 2021), but not in American cichlids. This is an important and new topic that should be studied further. These bones intervene in the dorsal flexion of the joints of the axial skeleton, when the neurocranial elevation occurs, to expand the oral cavity, at the time of suction feeding (Jimenez et al. 2018).

In the new species, there is no spinous process on the antero-superior border of the parahypural bone (Fig. 5a) of the caudal skeleton (vs. PP with a small spine in *Astronotusocellatus* and *A.crassipinnis*), and the elements of the hypural complex in *A.mikoljii* sp. nov., are more separated and are a little less robust than in the other two species (Fig. 5b, c). In addition, some spaces such as the diastema (D) are wider in *A.mikoljii* sp. nov. than in *A.crassipinnis* and *A.ocellatus*). Monod (1968), Sibilia and Andreata (1991), and Thieme et al. (2022) described all bones of the hypural complex of *A.ocellatus* as robust and less spaced. Elements such as E2 and E3 are represented as very close and even fused by these authors, something observed in the material examined (Fig. 5b, c), unlike *A.mikoljii* sp. nov. where these elements are more separated (Fig. 5a). Also, the E2 bone in *A.mikoljii* sp. nov. is articulated with the NEU2 spine, whereas in *A.ocellatus* they do not come into contact (Fig. 5b, c).

The analysis of the morphology of the sagitta otolith supports the distinctive taxonomic status of several fish species (Mereles et al. 2021, Reichenbacher et al. 2007, 2009a, b). However, the morphology of the sagitta otoliths was never used in previous studies for *Astronotus* species. Here, we compared the sagitta otoliths of *A.ocellatus* and *A.crassipinnis* (Figs 6, 10). The morphology of the sagitta otolith in *A.mikoljii* sp. nov. has an oval shape with strongly crenulated ventral and dorsal margins, while that in *A.crassipinnis* is elliptical with smooth-lobed margins and in *A.ocellatus*, it is elliptical with smooth-dentate margins. The rostrum of the sagitta otolith also showed differences: in *A.mikoljii* sp. nov. it is projected and larger than in *A.crassipinnis* and *A.ocellatus*; on the other hand, the posterior region of the sagitta otolith is rounded in *A.mikoljii* but straight or flat in *A.crassipinnis* and *A.ocellatus*. The biometric index and the morphometric index of sagitta otoliths in *A.mikoljii* sp. nov. also showed significant differences compared to *A.ocellatus* and *A.crassipinnis*. These results show that these species are clearly different in relation to the morphology of the sagitta otolith of *A.mikoljii* sp. nov. and the results of the genetic analysis showed the complete segregation of *A.mikoljii* sp. nov. from the other two recognized species of *Astronotus* (Fig. 9, Tables 4, 5).

In the Neotropical ichthyological literature there are several examples of species that initially had a pan-Neotropical or pan-Amazon distributions, which were later segregated into several species, including the genus *Cichla* Bloch & Schneider, 1801 (Kullander and Ferreira 2006). Likewise, Buitrago-Suárez and Burr (2007) described seven new species of *Pseudoplatystoma* Bleeker, 1862 from two species, which were previously considered to have a pan-Amazonian distribution, and Escobar et al. (2019) described a new species of *Piaractus* Eigenmann, 1903 from the Orinoco River basin, from a species previously thought widely distributed in the Amazon basin. More recently, Loboda et al. (2021) described two new species of freshwater rays of the genus *Paratrygon* Duméril 1865 from the Orinoco River basin from a single species with a shared distribution for the Orinoco and Amazon river basins.

Kullander (1986) and Colatreli et al. (2012) considered that there may be several species within the genus *Astronotus*, and particularly Colatreli et al. (2012) tested this hypothesis by using the ABGD method, delimiting five different lineages in *Astronotus* populations from the Amazon River basin that could represent candidate species. Our results using five distinct single-locus species delimitation methods also corroborate the findings of Colatreli et al. (2012) and indicate *A.mikoljii* sp. nov. as a novel and distinct lineage, more related to *Astronotus* sp. “Negro”, *A.crassipinnis*, and *Astronotus* sp. “East” lineages than to *A.ocellatus* (Fig. 9, Suppl. material 3: Data S1). Although the maximum credibility tree was able to recover two main clades of *Astronotus* with high posterior probability support, relationships among the lineages of the *A.crassipinnis* clade could not be fully resolved.

Considering that the new species is described from the Orinoco basin and that this basin is connected with the Negro basin by the Casiquiare channel, the strategy of including the Colatreli et al. (2012) sequences of individuals from the Negro River was essential for comparisons between these lineages and discussing the possibility of their being the same species. Thus, our delimitation analyses corroborate the findings of Colatreli et al. (2012) that the Negro River drainage has a distinct lineage, being also delimited by all the five methods used in this study. This lineage is probably *Astronotusrubroocellata* (originally *Cyclarubro-ocellata* Jardine & Schomburgk, 1843 in Schomburgk 1843): however, we emphasize that the revalidation of this species is beyond the scope of this study and should be the object of future studies.

Phylogenetic resolutions between *Astronotus* lineages/species were mostly well supported. Two large clades are observed, one formed by *A.ocellatus* and the *Astronotus* sp. “Jurua” lineage, and the other formed by *A.mikoljii* sp. nov. having as sister groups the *Astronotus* sp. “Negro”, *A.crassipinnis*, and *Astronotus* sp. “East” lineages. Despite the monophyly of all lineages/species being well supported, the phylogenetic relationships of these last three lineages showed low support values, which prevent us from discussing their relationships. It is clear that *A.mikoljii* sp. nov. is both genetically and morphologically differentiated from all other species/lineages within the genus and merits the status of a valid species. Although interspecific genetic distance is low (Table 4), other studies suggested that the low genetic distance pattern found in other Neotropical fish groups is indicative of recent diversification (Toffoli et al. 2008; Andrade et al. 2017).

## Supplementary Material

XML Treatment for Astronotus mikoljii

## Supplementary materials

Supplementary material 1 Table S1–S5 Data type Description of biometric indices used in otoliths Explanatory note Ratio and shape index used in morphometric analysis of otoliths from species of genus *Astronotus*. https://binary.pensoft.net/file/716305This dataset is made available under the Open Database License (http://opendatacommons.org/licenses/odbl/1.0/). The Open Database License (ODbL) is a license agreement intended to allow users to freely share, modify, and use this Dataset while maintaining this same freedom for others, provided that the original source and author(s) are credited.Alfredo Perez, Oscar M. Lasso-Alcalá, Pedro S. Bittencourt, Donald C. Tap­horn, Nayibe Perez, Izeni Pires Farias

Supplementary material 2 Figure S1 Data type Phylogenetic cladogram of all *Astronotus* species Explanatory note Neighbour Joining phylogenetic tree showing relationships among all 102 COI sequences of *Astronotus* plus five *Cichlaocellaris* sequences as outgroup. https://binary.pensoft.net/file/716306This dataset is made available under the Open Database License (http://opendatacommons.org/licenses/odbl/1.0/). The Open Database License (ODbL) is a license agreement intended to allow users to freely share, modify, and use this Dataset while maintaining this same freedom for others, provided that the original source and author(s) are credited.Alfredo Perez, Oscar M. Lasso-Alcalá, Pedro S. Bittencourt, Donald C. Tap­horn, Nayibe Perez, Izeni Pires Farias

Supplementary material 3 Data S1 Data type Metadata for all the sequences DNA used in this study are presented as a spreadsheet format xls, following the standard Darwin core format Explanatory note Metadata for all sequences generated in this study, sequences obtained from Colatreli et. al. (2012), and sequences downloaded from the GenBank nucleotide database. https://binary.pensoft.net/file/716307This dataset is made available under the Open Database License (http://opendatacommons.org/licenses/odbl/1.0/). The Open Database License (ODbL) is a license agreement intended to allow users to freely share, modify, and use this Dataset while maintaining this same freedom for others, provided that the original source and author(s) are credited.Alfredo Perez, Oscar M. Lasso-Alcalá, Pedro S. Bittencourt, Donald C. Tap­horn, Nayibe Perez, Izeni Pires Farias
