# Supplementary material for: ﻿A new species of Astronotus (Teleostei, Cichlidae) from the Orinoco River and Gulf of Paria basins, northern South America
Source: Zookeys. 2022 Jul 18;1113:111–52. doi: 10.3897/zookeys.1113.81240 (PMC9848875; doi:10.3897/zookeys.1113.81240)
Supplement: Supplementary material 1 — Table S1–S5 [file zookeys-1113-111_article-81240__-s001.docx]

**Supplementary material 1**

**Table S1.** Authors: Alfredo Perez, Oscar M. Lasso-Alcalá, Pedro S. Bittencourt, Donald C. Taphorn, Nayibe Perez, Izeni Pires Farias

Data type: Description of biometric indices used in otoliths

Explanatory note: Ratio and shape indices used in morphometric analysis of sagitta otoliths from species of the genus *Astronotus*

| **Biometric index** | **Ratio** | **Description** | **References** |
| --- | --- | --- | --- |
| Sulcus-otolith ratio | SUL/OL | Proportion of (SUL) contained in the (OL) | Avigliano et al. (2016) |
| Ostium - sulcus ratio | OCL/SUL | Proportion of the (OCL) contained in the (SUL) | Avigliano et al. (2016) |
| Ostium - cauda ratio | OCL/CCL | Proportion of the (OCL) contained in the (CCL) | Schwarzhans (1993) |
| Ostium width- rostrum width ratio | OCW/RW | Proportion of the (OCW) contained in the (RW) | Using for the first time |
| Colliculum width- rostrum width ratio | CCW/RW | Proportion of the (CCW) of the contained in the (RW) | Using for the first time |
| Rostrum width- post-rostrum distance | RW/PRD | Proportion of the contained in the (PRD) | Using for the first time |
| Rostrum/otolith length ratio | RL/OL | Proportion of the (RL) contained in the (OL) | Volpedo and Echeverría (2000). |
| **Shape index** | **Equation** | **Description** | **References** |
| Aspect ratio | Ar =  OL/OW | Expresses the shape of the otolith for an elongated or circular shape. | Tuset et al. (2006) |
| Rectangularity | RT =  AO/OL*OW) | Describe the variation of Otolith Length (OL) and Otolith Width (OW), in relation to the area (AO). | Bani et al. (2013) |
| Form factor | FF =  (4πAO)/Po^2^) | Evaluates the irregularity of the surface area (AO) of the Otolith’s Perimeter (Po). | Bani et al. (2013) |
| Roundness | Rd =  (4AO)/(πOL^2^) | Demonstrates variation of otolith from a perfect circle. | Bani et al. (2013) |
| Complexity | Dp =  Po/2*(√(AO*π) | Expresses the complexity (protuberances and grooves vs. smooth) of the edge of the otoliths | Kalff (2002) |

**Table S2.** Authors: Alfredo Perez, Oscar M. Lasso-Alcalá, Pedro S. Bittencourt, Donald C. Taphorn, Nayibe Perez, Izeni Pires Farias

Data type: Result of the ANOVA-one way of the biometric index.

Explanatory note: Comparison of biometric and shape index from sagitta otoliths of the species of *Astronotus* with mean (*X*); standard deviation (SD); Fisher test (*F*); significance level (*P* = 0.05).

| **Species** |  | ***A. crassipinnis*** *n =* 23 | ***A. mikoljii* sp. nov.**  *n =* 18 | ***A. ocellatus***  *n =* 10 | ***F*** | ***P* = 0.05** |
| --- | --- | --- | --- | --- | --- | --- |
| **Biometric index** |  |  |  |  |  |  |
| SUL/OL | *X* | 0.905 | 0.896 | 0.918 | 6.410 | 0.003* |
|  | SD | 0.016 | 0.013 | 0.015 |  |  |
| OW/OL | *X* | 0.152 | 0.154 | 0.147 | 0.588 | 0.559 |
|  | SD | 0.017 | 0.011 | 0.012 |  |  |
| RW/OL | *X* | 0.291 | 0.299 | 0.263 | 5.560 | 0.006* |
|  | SD | 0.027 | 0.021 | 0.038 |  |  |
| OCL/OL | M | 0.379 | 0.362 | 0.383 | 1.866 | 0.165 |
|  | SD | 0.035 | 0.032 | 0.024 |  |  |
| LR/OL | *X* | 0.168 | 0.175 | 0.168 | 0.238 | 0.788 |
|  | SD | 0.032 | 0.031 | 0.034 |  |  |
| OW/RW | *X* | 0.521 | 0.517 | 0.569 | 2.743 | 0.074 |
|  | SD | 0.056 | 0.058 | 0.077 |  |  |
| CCW/RW | *X* | 0.358 | 0.347 | 0.370 | 0.736 | 0.484 |
|  | SD | 0.052 | 0.045 | 0.047 |  |  |
| RW/PRD | *X* | 0.367 | 0.377 | 0.335 | 2.697 | 0.077 |
|  | SD | 0.044 | 0.038 | 0.063 |  |  |
| OW/PRD | *X* | 0.735 | 0.837 | 0.767 | 15.37 | 0.000* |
|  | SD | 0.037 | 0.068 | 0.078 |  |  |
| **Shape index** |  |  |  |  |  |  |
| Dp | *X* | 1.279 | 1.282 | 1.307 | 1.344 | 0.270 |
|  | SD | 0.038 | 0.049 | 0.059 |  |  |
| Ar | *X* | 0.585 | 0.665 | 0.606 | 19.84 | 0.000* |
|  | SD | 0.027 | 0.049 | 0.049 |  |  |
| Rd | *X* | 0.543 | 0.597 | 0.545 | 9.848 | 0.003* |
|  | SD | 0.029 | 0.055 | 0.032 |  |  |
| RT | *X* | 0.728 | 0.703 | 0.706 | 6.802 | 0.002* |
|  | SD | 0.016 | 0.028 | 0.023 |  |  |
| Ff | *X* | 0.612 | 0.610 | 0.587 | 1.241 | 0.298 |
|  | SD | 0.035 | 0.046 | 0.050 |  |  |

**Table S3.** Authors: Alfredo Perez, Oscar M. Lasso-Alcalá, Pedro S. Bittencourt, Donald C. Taphorn, Nayibe Perez, Izeni Pires Farias.

Data type: Meristic values of the dorsal and vertebral skeleton

Explanatory note: Number of elements of the dorsal and vertebral skeleton between the three species of *Astronotus*, *A. mikoljii* sp. nov., *A. ocellatus*, *A. crassipinnis*.

|  | ***A*. *mikoljii* sp. nov.**  *n* = 26 | ***A. ocellatus***  *n* = 10 | ***A. crassipinnis***  *n* = 4 |
| --- | --- | --- | --- |
| Meristic variable | mode (min - max) | mode (min - max) | mode (min - max) |
| Supraneural bones | II (II - III) | II (II) | II (II) |
| Precaudal vertebrae | 15 (14 - 16) | 15 | 15 - 16 |
| Caudal vertebrae (urostyle included ) | 17 (15 - 18) | 17 (16 - 17) | 17 (16 - 17) |
| Total vertebrae | 32 (30 - 33) | 32 (31 - 32) | 32 - 33 |

**Table S4.** Authors: Alfredo Perez, Oscar M. Lasso-Alcalá, Pedro S. Bittencourt, Donald C. Taphorn, Nayibe Perez, Izeni Pires Farias.

Data type: Meristic values of the hypural complex

Explanatory note: Number of rays and their position in the caudal skeleton (hypural complex) between the three species of *Astronotus*, with following descriptors: hemal spine (HEM2-3); parahypural (PH); hypaxial procurrent caudal rays (HPCR); hypaxial caudal rays (HCR); epaxial caudal rays (ECR); epaxial procurrent caudal rays (EPCR); total caudal rays (TCR); hypurals (HI–V); epurals (E1–2); neural spine (NEU2–3).

| **Caudal skeleton** | ***A*. *mikoljii* sp. nov.**  *n* = 26 | ***A*. *ocellatus***  *n* = 10 | ***A. crassipinnis***  *n* = 4 |
| --- | --- | --- | --- |
| **Meristic variable** | **mode (min - max)** | **mode (min - max)** | **mode (min - max)** |
| HEM3 / HPCR | 1 (1 - 2) | 1 (1 - 2) | 1 - 2 |
| HEM2 / HPCR | 1 (0 - 2) | 2 (1 - 2) | 1 (1 - 3) |
| HEM2 / HCR | 1 (1 - 2) | 1 | 1 |
| PH / HCR | 3 (2 - 4) | 3 (2 - 4) | 2 -3 |
| HI / HCR | 3 (2 - 4) | 3 (2 - 3) | 3 (3 - 4) |
| HII / HCR | 1 (1 - 2) | 1 - 2 | 1 (1 - 2) |
| HIII / ECR | 2 (2 - 3) | 2 | 2 (1 - 2) |
| HIV / ECR | 3 (2 - 4) | 4 (3 - 4) | 3 - 4 |
| HV / ECR | 2 (1 - 3) | 2 (2 - 3) | 3 (2 - 3) |
| E2 / ECR | 0 (0 - 1) | 0 (0 - 1) | 0 |
| E2 / EPCR | 1 | 1 | 1 (1 - 2) |
| E1 / EPCR | 1 | 1 | 2 (1 - 2) |
| NEU3 / EPCR | 1 (0 - 2) | 1 (1 - 2) | 1 (0 -1) |
| HPCR | 3 (2 - 3) | 3 (2 - 4) | 3 (3 - 4) |
| HCR | 8 (8 - 10) | 8 | 8 |
| ECR | 8 (7 - 8) | 8 | 8 |
| EPCR | 3 (2 - 4) | 3 (2 - 4) | 3 (3 - 4) |
| TCR | 22 (20 - 24) | 22 (21 - 23) | 22 (22 - 24) |

**Table S5.** Authors: Alfredo Perez, Oscar M. Lasso-Alcalá, Pedro S. Bittencourt, Donald C. Taphorn, Nayibe Perez, Izeni Pires Farias

Data type: Results of the confusion matrix from biometric indices of the sagitta otoliths

Explanatory note: Classification matrix as a result of Canonical Discriminant Analyses (CDA) between the three species of *Astronotus.*

|  | ***A. crassipinnis*** | ***A. mikoljii* sp. nov.** | ***A. ocellatus*** | **Total** |
| --- | --- | --- | --- | --- |
| ***A. crassipinnis*** | 21 | 0 | 2 | 23 |
| ***A. mikoljii* sp. nov.** | 0 | 18 | 0 | 18 |
| ***A. ocellatus*** | 1 | 0 | 8 | 9 |
| **Total** | 22 | 18 | 10 | 50 |

**References**

Avigliano E, Fortunato RC, Biolé F, Domanico A, De Simone S, Neiff JJ, Volpedo AV (2016) Identification of nurseries areas of Juvenile *Prochilodus lineatus* (Valenciennes, 1836) by scale and otolith morphometry and microchemistry. Neotropical ichthyology 14 (3): e160005. [https://dx.doi.org/10.1590/1982-0224-20160005](http://dx.doi.org/10.1590/1982-0224-20160005)

Bani A, Poursaeid S, Tuset VM (2013) Comparative morphology of the sagittal otolith in three species of south Caspian gobies. Journal of Fish Biology 82: 1321–1332. [https://dx.doi.org/10.1111/jfb.12073](https://doi.org/10.1111/jfb.12073)

Kalff J (2002) Limnology: inland water ecosystems Upper Saddle River, Prentice–Hall Publishing, New Jersey, 592 pp.

Schwarzhans W (1993) A comparative morphological treatise of Recent and fossil otoliths of the family Sciaenidae (Perciformes). Piscium Catalogas: Part Otolithi Piscium 1: 1–245.

Volpedo AV, Echeverría D (2000) Catálogo y claves de otolitos para la identificación de peces del Mar Argentino. 1. Peces de importancia comercial. Editorial Dunken, Buenos Aires, 90 pp.

Tuset VM, Rosin PL, Lombarte A. (2006) Sagittal otolith shape used in the identification of fishes of the genus *Serranus*. Fisheries Research. 81(2–3): 316-325. <https://dx.doi.org/10.1016/j.fishres.2006.06.020>
