# Supplementary material for: ﻿A new species of Astronotus (Teleostei, Cichlidae) from the Orinoco River and Gulf of Paria basins, northern South America
Source: Zookeys. 2022 Jul 18;1113:111–52. doi: 10.3897/zookeys.1113.81240 (PMC9848875; doi:10.3897/zookeys.1113.81240)
Supplement: Supplementary material 2 — Figure S1 [file zookeys-1113-111_article-81240__-s002.docx]

**Supplementary material 2**

**Figure S1.** Authors: Alfredo Perez, Oscar M. Lasso-Alcalá, Pedro S. Bittencourt, Donald C. Taphorn, Nayibe Perez, Izeni Pires Farias

Data type: Phylogenetic Cladogram of All *Astronotus* species

Explanatory note: Neighbor-joining phylogenetic tree. Phylogenetic tree showing all 102 COI sequences of *Astronotus*, constructed from p-distances, plus five *Cichla ocellaris* sequences as an outgroup.

| 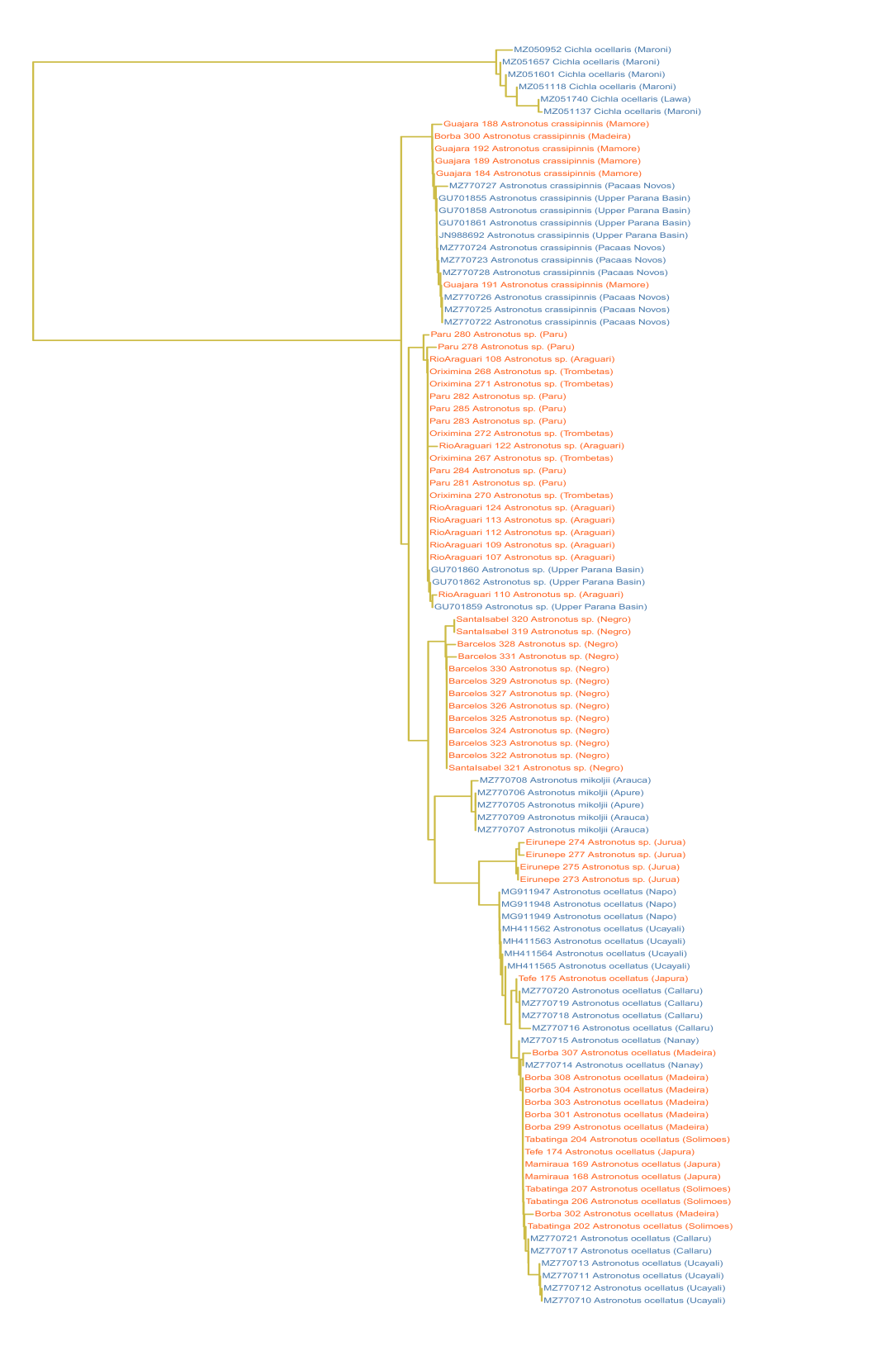 |
| --- |
